# Integrated multi-omics identifies dysregulated lipid metabolism of paresis in dairy sheep during the early transition period

**DOI:** 10.1128/spectrum.01544-25

**Published:** 2025-10-09

**Authors:** Shuai Jiao, Fei Li, Tianxi Zhang, Guojie Yang, Ronghui Lu, Fadi Li, Long Guo, Zhiyuan Ma, Pengshan Zhao, Baocang Liu

**Affiliations:** 1 State Key Laboratory of Grassland Agro-ecosystems, Key Laboratory of Grassland Livestock Industry Innovation, Ministry of Agriculture and Rural Affairs, Engineering Research Center of Grassland Industry, Ministry of Education, College of Pastoral Agriculture Science and Technology, Lanzhou University 12426, Lanzhou, China; 2 Gansu Yuansheng Agriculture and Animal Husbandry Technology Co., Ltd., Jinchang, China; 3 Xinjiang Taikun Group Changji Feed Co., Ltd., Changji, China; University of Arkansas Fayetteville, Fayetteville, Arkansas, USA

**Keywords:** dairy sheep, biomarkers, paresis, multi-omics, transition period

## Abstract

Paresis during the early transition period is a prevalent metabolic disorder in prolific dairy sheep, characterized by a complex and poorly understood pathogenesis. This study longitudinally monitored a cohort of dairy sheep from 21 days antepartum to 1 day postpartum. During this period, blood, fecal, and colostrum samples were collected. The pathway of paresis was revealed by plasma metabolome and fecal 16S sequencing, and potential early biomarkers were identified. The physiological parameters of healthy dairy sheep (HDS) and paretic dairy sheep (PDS) differed in both the antepartum (not yet paresis) and postpartum (paresis) periods. Elemental analysis revealed higher levels of copper, potassium, and magnesium in PDS colostrum compared to HDS. Metabolomic analysis of HDS and PDS in antepartum identified 37 differential metabolites, with acylcarnitines (3-hydroxyhexadecadienoylcarnitine and 3-hydroxyoctanedioylcarnitine) emerging as promising early diagnostic biomarkers. 16S rRNA sequencing revealed distinct microbial signatures, with genera such as *Fusobacterium* and *Erysipelatoclostridium* enriched in PDS, whereas *Faecalibacterium* and *Bacillus* were more abundant in HDS. Integration of multi-omics data in postpartum revealed differences in glycerophospholipid metabolism, phenylalanine, tyrosine and tryptophan biosynthesis, glycine, serine and threonine metabolism, and primary bile acid biosynthesis between PDS and HDS. Our findings suggest that periparturient paresis in dairy sheep is linked to abnormal lipid and amino acid metabolism, with early potential biomarkers such as acylcarnitines identified. This study provides critical insights for developing strategies to prevent and manage periparturient paresis through targeted nutritional interventions and disease control.

**IMPORTANCE:**

This study investigates paresis in dairy sheep during the early transition period, identifying metabolic and physiological markers for early diagnosis. Longitudinal monitoring revealed prepartum differences in glucolipid profiles, liver enzymes, and oxidative stress markers between healthy and paretic sheep. Metabolomics identified 37 antepartum differential metabolites, including acylcarnitines, as potential biomarkers. Gut microbiota analysis revealed genera such as *Fusobacterium* and *Erysipelatoclostridium* enriched in paretic sheep, and *Faecalibacterium* and *Bacillus* in healthy individuals. Postnatal integration of multi-omics data revealed that paresis is closely associated with lipid metabolism and amino acid metabolism in dairy sheep. These findings support targeted nutritional strategies to mitigate periparturient metabolic disorders, enhancing dairy sheep health and productivity.

## INTRODUCTION

Approximately 85% of global milk production originates from cattle, with buffalo, goat, sheep, and camel milk contributing 11%, 2.3%, 1.4%, and 0.2%, respectively (1). Among these, sheep milk is highly nutritious, with an average protein content of 5.5 g/100 g, which is 1.6 times higher than cow milk and 1.5 times higher than goat milk. Additionally, its fat content averages 5.9%, surpassing cow milk by 1.8 times and goat milk by 1.6 times (2, 3). Therefore, it is essential to enhance the breeding, nutrition, feeding, and management practices for dairy sheep, alongside advancing the local dairy sheep industry to meet the growing public demand.

During the transition period, the changes in metabolism, physiological conditions, and inflammatory states increase susceptibility to diseases such as mastitis, metritis, and metabolic diseases (4, 5). Paresis may be defined as “a deficiency in the generation of the gait or in the ability to support weight” and implies that a degree of voluntary movement is still present (6). Parturient paresis in sheep and goats typically occurs in outbreaks during the last weeks of gestation, affecting usually <5% but up to 30% in severe cases (7). The primary causes of paresis in dairy sheep include imbalances in calcium and phosphorus, pregnancy toxemia, insufficient exercise, and other disease-related factors (6). Pregnancy toxemia, a major contributor to paresis, has a poor prognosis, with a reported case fatality rate of 86% in dairy goats (8, 9). The central metabolic events of pregnancy toxemia are fat mobilization and the availability of glucose, typically triggered by negative energy balance (NEB) during late gestation, when energy demands for fetal growth exceed intake capacity (10, 11). Obese and sedentary pregnant ruminants face compounded challenges due to reduced ruminal space caused by intra-abdominal fat and an expanding uterus, further exacerbating the NEB (12). In cases of hypocalcemia, decreased phosphocreatine expression results in reduced adenosine triphosphate production in muscles, leading to paresis in dairy cows (13). In summary, the development of paresis is intricately linked to disorder in energy and fat metabolism.

The gastrointestinal tract is a highly complex ecosystem, where the dynamic interactions among diet, microbiota, and host tissues play a fundamental role in shaping the development and physiological health (14). Accumulating evidence indicates that metabolic disorders such as obesity and diabetes are closely linked to gut microbial dysbiosis, which alters both microbial composition and function (15, 16). Beyond its metabolic roles, the gut microbiota is also a key regulator of host immunity. It contributes to immune system maturation and homeostasis, while microbial perturbations can result in immune dysregulation and chronic inflammation (17, 18). Although paresis, a metabolic disorder, has been associated with disruptions in metabolism and immune function, the gut microbial alterations in paretic dairy sheep (PDS) vs healthy dairy sheep (HDS), as well as their potential mechanistic contributions, remain largely uncharacterized.

Blood metabolites are valuable biomarkers for diagnosing animal diseases and monitoring metabolic states (19). Livestock metabolomics has advanced the identification of disease-associated biomarkers across various species (20, 21). For example, Hailemariam et al. (22) identified carnitine, propionyl carnitine, and lysophosphatidylcholine acyl C14:0 as plasma biomarkers in dairy cows, capable of predicting disease states up to 4 weeks before clinical onset. Similarly, Uzti̇mür and Ünal (23) reported that hormones such as spexin (decreased by 56.1 pg/mL) and irisin (decreased by 0.51 µg/mL) demonstrated high sensitivity, specificity, and diagnostic performance for pregnancy toxemia, with area under the curve (AUC) values of 0.988 and 0.979, respectively. In short, multi-metabolite panels hold significant potential for predicting periparturient diseases. The energy requirement of a pregnant ewe carrying twins or triplets is 180% or 240% greater, respectively, than that of an ewe with a single fetus during the final month of gestation (24). Crossbreeding East Friesian sheep with Hu sheep improves adaptation to local breeding conditions, enhances lactation efficiency, and produces highly prolific crossbreds with lambing rates of up to 230%. However, this also heightens their susceptibility to pregnancy toxemia and subsequent paresis. Therefore, leveraging omics technologies to identify early biomarkers and elucidate the metabolic pathways associated with paresis is of paramount importance.

In light of the metabolic changes linked to paresis, we hypothesize that specific blood metabolites and microbial signatures in the perinatal period are associated with the occurrence of paresis. This study aims to compare plasma physiological parameters and blood–milk element profiles between healthy and paretic dairy sheep, revealing distinct metabolomic and microbiome characteristics that may help identify early biomarkers and inform future strategies for prevention and management.

## MATERIALS AND METHODS

### Animals, diets, and experimental design

The experiment was conducted at the Yuansheng First Ecological Ranch, which is part of Gansu Yuansheng Agricultural Technology Co., Ltd., located in Yongchang County, Gansu Province (38°27′N, 102°07′E). A total of 156 hybrid dairy sheep (East Friesian sheep males × Hu sheep females) in the early transition period (about 21 days in antepartum) were selected from a population of 11,708 dairy sheep. From the early transition period to the postpartum period, 122 ewes lambed successfully, excluding cases of mortality or non-pregnancy. Paresis was defined as “a deficiency in the generation of the gait or in the ability to support weight” while retaining partial voluntary movement (6). Affected sheep exhibited characteristic clinical signs, including stiff gait, ataxia, salivation, constipation, and muscle tremors. Diagnostic criteria were established according to the clinical descriptions and video documentation of periparturient paresis in Crilly et al. (6).

**Fig 1:**
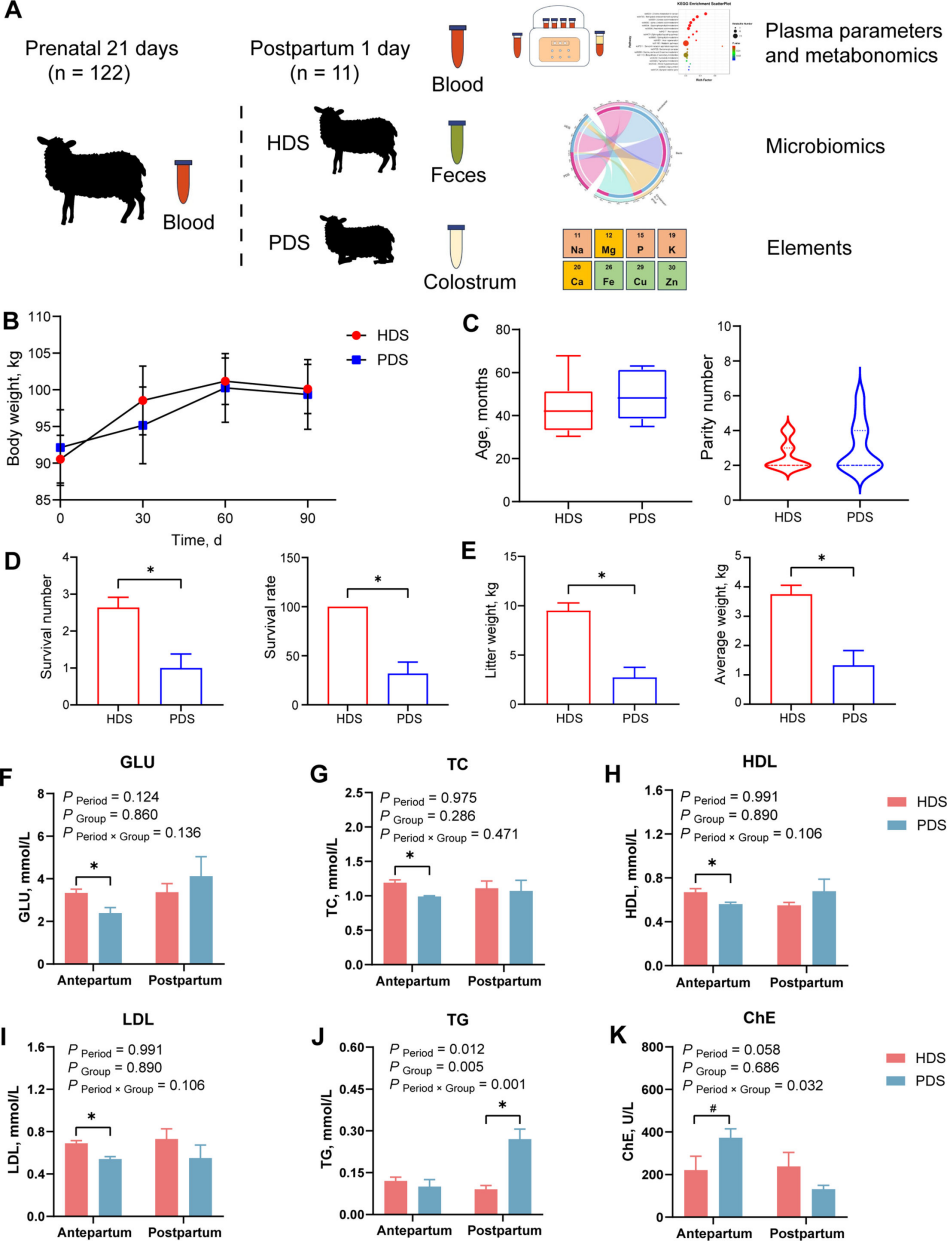
Phenotypic characteristics and plasma glucolipid metabolism of healthy and paretic dairy sheep at different periods. (**A**) Study and sampling design of the dairy sheep trial. (**B**) Maternal body weight at 0, 30, 60, and 90 days of pregnancy in the HDS and PDS groups. (**C**) Age and parity in HDS and PDS groups. (**D**) Survival number and survival rate of offspring in HDS and PDS groups. (**E**) Birth litter weight and average weight of surviving offspring in HDS and PDS groups. (**F–K**) Plasma glucolipid metabolism of HDS and PDS groups in antepartum and postpartum. GLU, glucose; TC, cholesterol; HDL, high-density lipoprotein; LDL, low-density lipoprotein; TG, triglyceride; ChE, cholinesterase; HDS, healthy dairy sheep; PDS, paretic dairy sheep. *, significantly different between the two groups (*P* < 0.05). #, a tendency to differ between the two groups (0.05 < *P* < 0.10).

Among these, 11 dairy sheep experienced paresis, with onset occurring between 3 days prepartum and 1 day postpartum. We selected HDS (*n* = 11) and PDS (*n* = 11) from 122 dairy sheep without antibiotics or drug treatment (Fig. 1A). HDS were selected from non-paretic individuals matched to PDS cases by age, parity, and body weight. All HDS demonstrated normal feed intake, alert mentation, and absence of neurological/musculoskeletal abnormalities. The two groups were defined at 21 days antepartum (not yet paresis) as APDS (paretic dairy sheep in antepartum) and AHDS (healthy dairy sheep in antepartum). Throughout the experimental period, all dairy sheep were group-housed and managed under uniform conditions, with consistent provision of diet, water, and environmental factors. The dairy sheep were fed a basal total mixed ration (TMR; Table S1). The TMR was provided twice daily via an automatic feed wagon to ensure *ad libitum* intake, at approximately 7:00 a.m. and 6:00 p.m.

### Sample collection

Blood samples were collected into EDTA vacutainer tubes from the jugular vein at 7:00 a.m., 21 days before delivery, and 1 day after delivery. Samples were centrifuged at 3,000 × *g* at 4°C for 15 min, and the plasma was collected. Plasma samples were frozen in liquid nitrogen and stored at −80°C for subsequent analysis. Before feeding on the morning of 1 day in postpartum, fecal samples were manually collected from the ewe’s rectum using sterile gloves. Fecal samples were transferred to sterile 5 mL cryogenic tubes, immediately quenched in liquid nitrogen, and stored at −80°C for later analysis. Colostrum samples were collected into 50 mL centrifuge tubes after feeding on the morning of the second day postpartum and stored at −20°C for later analysis.

### Plasma parameter measurement

Plasma parameters were measured by colorimetric method using the Mindray BS-420 automatic biochemical analyzer (Shenzhen Mindray Biomedical Electronics Co., Ltd., Shenzhen, China). The levels of total protein (TP), blood urea nitrogen (BUN), creatinine (CREA), glucose (GLU), cholesterol (TC), high-density lipoprotein (HDL), low-density lipoprotein (LDL), triglyceride (TG), cholinesterase (ChE), albumin (ALB), alanine aminotransferase (ALT), aspartate aminotransferase (AST), total bilirubin (TBIL), creatine kinase (CK), alkaline phosphatase (ALP), and lactate dehydrogenase (LDH) were detected using commercially available detection kits (Zhongsheng Beikong Bio-Technology and Science Inc., Beijing, China). Immunoglobulin A (IgA), immunoglobulin G (IgG), and immunoglobulin M (IgM) were detected using detection kits (Beijing SINO-UK Institute of Biological Technology, Beijing, China). Total antioxidant capacity (T-AOC), superoxide dismutase (SOD), catalase (CAT), glutathione peroxidase (GSH-Px), and malondialdehyde (MDA) levels were detected using commercially available detection kits (Nanjing Jiancheng Bioengineering Institute, Nanjing, China) according to the manufacturer’s instructions.

### Element determination

Plasma and colostrum samples were thoroughly mixed after thawing. A 1 mL sample was added to the microwave digestion tank, followed by 6 mL of nitric acid and 2 mL of hydrogen peroxide. The rubber pad was then placed, left to stand for 30 min, and the outer cover was tightened. Samples were placed in a microwave digestion instrument (Tank 40, Xinyi Microwave Chemical Technology Co., Ltd., Shanghai, China), with digestion temperatures varying between samples. For plasma samples, the temperature was ramped for 12 min to 180°C, with a holding time of 10 min. For colostrum samples, the temperature was ramped for 12 min to 190°C, with a holding time of 10 min. After digestion, the acid was expelled using an acid expeller (BHW-09A45, Botong Chemical Technology Co., Ltd., Shanghai, China), and the temperature was set to 160°C. The digestion solution was transferred to a 50 mL volumetric flask and diluted with ultrapure water. The solution was then filtered through a 0.22 µm membrane and stored at 4°C for testing. Standard solutions of calcium (Ca), copper (Cu), iron (Fe), potassium (K), magnesium (Mg), sodium (Na), and zinc (Zn) were prepared using an inorganic element mixed solution reference material (GBW(E)081531, National Institute of Metrology, Beijing, China). A single-element standard solution was used to determine phosphorus (GSB 04-1741-2004, National Center for Analysis and Testing of Nonferrous Metals and Electronic Materials, Beijing, China). Trace elements were determined using an inductively coupled plasma optical emission spectrometer (5800 ICP-OES, Agilent Technologies Inc., USA).

### Non-targeted metabolomics

Plasma samples were thawed on ice, and metabolites were extracted using an 80% methanol buffer. Briefly, 100 µL of the sample was transferred to a 2.0 mL Eppendorf (EP) tube, and 400 µL of ice-cold methanol solution was added and mixed thoroughly. The extraction mixture was stored at −20°C for 30 min to precipitate proteins. After centrifugation at 20,000 × *g* for 15 min, 400 µL of the supernatant was transferred to a new EP tube. The supernatant was centrifuged again and transferred into an injection vial for ultra-performance liquid chromatography-high resolution mass spectrometry (UPLC-HRMS) analysis. Additionally, 15 µL of the extract from each sample was pooled to create a quality control sample. Chromatographic separations were conducted using an UltiMate 3000 UPLC system (Thermo Fisher Scientific, Bremen, Germany). An Acquity UPLC T3 column (100 mm × 2.1 mm, 1.8 µm; Waters, Milford, USA) was used for analysis. The high-resolution mass spectrometer used for collection was Q-Exactive (Thermo Fisher Scientific, Bremen, Germany). Each sample was analyzed in both positive and negative ionization modes.

Preprocessing of mass spectrometry (MS) data, including peak detection, clustering, retention time alignment, secondary clustering, and annotation of isotopes and adducts, was performed using the XCMS software suite (25). The raw liquid chromatography-mass spectrometry (LC-MS) data files were initially converted into the mzXML format. This conversion facilitated subsequent analysis using the XCMS, CAMERA, and metaX toolboxes, all of which are integrated within the R software environment (26, 27). Each ion was characterized by its retention time and mass-to-charge ratio (*m*/*z*). The intensities of the detected peaks were meticulously documented. Kyoto Encyclopedia of Genes and Genomes (KEGG) and Human Metabolome Database (HMDB) were used to annotate metabolites by aligning molecular mass (*m*/*z*) data from the samples with database entries. Annotation was considered successful if the mass discrepancy between experimental values and database entries was within 10 ppm.

Statistical analyses were conducted using R (version 4.0.0). Orthogonal partial least squares discriminant analysis (OPLS-DA) was performed using the metaX package and visualized with ggplot2. Differential metabolites were identified based on a *t*-test (*P* < 0.05) and variable importance in projection (VIP) > 1. Metabolites with a log_2_ fold change (log_2_FC) > 0 were considered upregulated, while those with log_2_FC < 0 were considered downregulated. Classification is performed using a standard Support Vector Machine model, and the receiver operating characteristic (ROC) curve is constructed. The AUC metric is used to evaluate the model’s performance. Based on the hypergeometric test, the differential enrichment analysis of KEGG Pathway was carried out. Functional categories with *P* < 0.05 were considered significantly enriched in differential metabolites. The clustering heat map is drawn by the R package pheatmap. Correlation analysis was conducted using Pearson correlation coefficients in the R package cor. Network diagrams were created based on the pathways associated with the metabolites.

### 16S amplicon sequencing

The total DNA of the microbial group was extracted using a magnetic bead fecal genomic DNA extraction kit (AU46111-96, BioTeke, China). Specific primers (341F: 5′-CCTACGGGNGGCWGCAG-3′ and 805R: 5′-GACTACHVGGGTATCTAATCC-3′) were selected, and PCR reagents were added according to the reaction system to amplify the 16S rRNA gene fragment in the V3-V4 region (28). The PCR program consisted of initial denaturation at 98°C for 30 s, followed by 32 cycles of denaturation (98°C for 10 s), annealing (54°C for 30 s), elongation (72°C for 45 s), and a final extension step (72°C for 10 min). The PCR products were purified by AMPure XT beads (Beckman Coulter Genomics, Danvers, MA, USA) and quantified by Qubit (Invitrogen, USA). The purified PCR products were evaluated using Agilent 2100 bioanalyzer (Agilent, USA) and Illumina library quantification kits (KapaBiosciences, Woburn, MA, USA). After that, 2 × 250 bp double-end sequencing was performed using a NovaSeq 6000 sequencer.

Raw sequencing data underwent primer excision with cutadapt (version 1.9) following de-multiplexing. Subsequently, paired-end reads were merged using FLASH (version 1.2.8). To enhance data quality, reads with low quality scores below 20, lengths shorter than 100 base pairs, or containing over 5% ambiguous “N” nucleotides were refined using the sliding-window algorithm in fqtrim (version 0.94). Subsequent quality filtering ensured the acquisition of pristine tags. Vsearch (version 2.3.4) was employed to eliminate chimeric sequences (29). DADA2 was then utilized for denoising and the derivation of amplicon sequence variants (ASVs) (30). For species annotation, sequence alignment was conducted using the feature-classifier plugin in QIIME2, with reference to the SILVA and NT-16S databases (31). Alpha diversity was evaluated using the Chao 1, Shannon, Simpson, and Ace indices, and statistical comparisons between groups were performed using the Wilcoxon rank-sum test. Beta diversity was assessed using Bray-Curtis dissimilarity and visualized with principal co-ordinates analysis plots. The assessment of alpha and beta diversities, as well as the determination of bacterial taxonomy based on relative abundance, was all executed through QIIME2. To discern differentially abundant genera, the Mann-Whitney *U* test was applied, with significance set at *P* value <0.05. The linear discriminant analysis (LDA) effect size (LEfSe; LDA ≥ 2.0, *P* < 0.05) was calculated using the nsegata-lefse package. Additional graphical representations were created with the R language (version 3.4.4). To understand differential microbial interactions, we constructed networks based on the relative abundance of genera. The genera correlation network within the PDS and HDS was analyzed by Spearman’s correlation coefficient in the R package Hmisc (version 4.6.0). The significant correlation (|rho| > 0.70 and adjusted *P* < 0.05) among different genera was visualized using Cytoscape version 3.8.2. Functional profiles of the microbial communities were predicted from 16S rRNA gene sequencing data using PICRUSt2 and annotated using the KEGG database.

### Statistical analysis

Basic information and colostrum elements of different groups of dairy sheep were analyzed using unpaired *t*-test of prism (GraphPad Software Inc., 8.0, La Jolla, CA, USA). In addition, plasma parameters and plasma element contents were analyzed using a two-way analysis of variance design using the MIXED procedure of SAS (SAS Enterprise Guide 5.1, SAS Institute Inc., Cary, NC, USA) according to the following model:


$$Y_{ijk}=\mu +P_{i}+G_{Gj}+(PG{)}_{ij}+e_{ijk},$$

where μ is the overall mean; *P*_*i*_ is the effect of period *i*; *G*_*j*_ is the effect of group *j*; (*PG*)_*ij*_ is the interaction between period *i* and group *j*; *e*_*ijk*_ is the random error. Data were presented as means and pooled standard error. Statistical significance was declared at *P* < 0.05 and tendencies at 0.05 ≤ *P*  ≤ 0.10.

## RESULTS

### Information of dairy sheep maternal and its offspring

The data for the overall herd are displayed in Table S2. The mean age of the dairy sheep maternal in the experiment was 39.30 ± 10.54 months, the mean parity was 2.18 ± 1.00, and the mean litter number was 2.33 ± 0.94. The average maternal weights at 0, 30, 60, and 90 days after pregnancy and 1 day after delivery were as follows: 87.63 ± 12.00 kg, 91.31 ± 13.14 kg, 96.61 ± 13.16 kg, 96.02 ± 12.80 kg, and 86.19 ± 12.78 kg. The mean survival number of offspring was 2.07 ± 1.04, the death number was 0.25 ± 0.62, and the mean survival rate was 89.21% ± 26.28%. The litter weight of surviving offspring was 7.65 ± 3.32 kg, and the average weight was 3.74 ± 1.53 kg.

There were no differences in the age, parity, and weight during pregnancy between PDS and HDS (*P* > 0.10; Fig. 1B and C). The survival number of offspring and the survival rate in HDS were higher than those in PDS (*P* < 0.05; Fig. 1D). In addition, the litter weight and average body weight of offspring in HDS were higher than those in PDS (*P* < 0.05; Fig. 1E). There was no difference in litter number between PDS and HDS, but the death number of offspring in HDS was lower than that in PDS (*P* < 0.05, Table S3).

### Glucolipid metabolism, protein metabolism, and liver function

**Fig 2:**
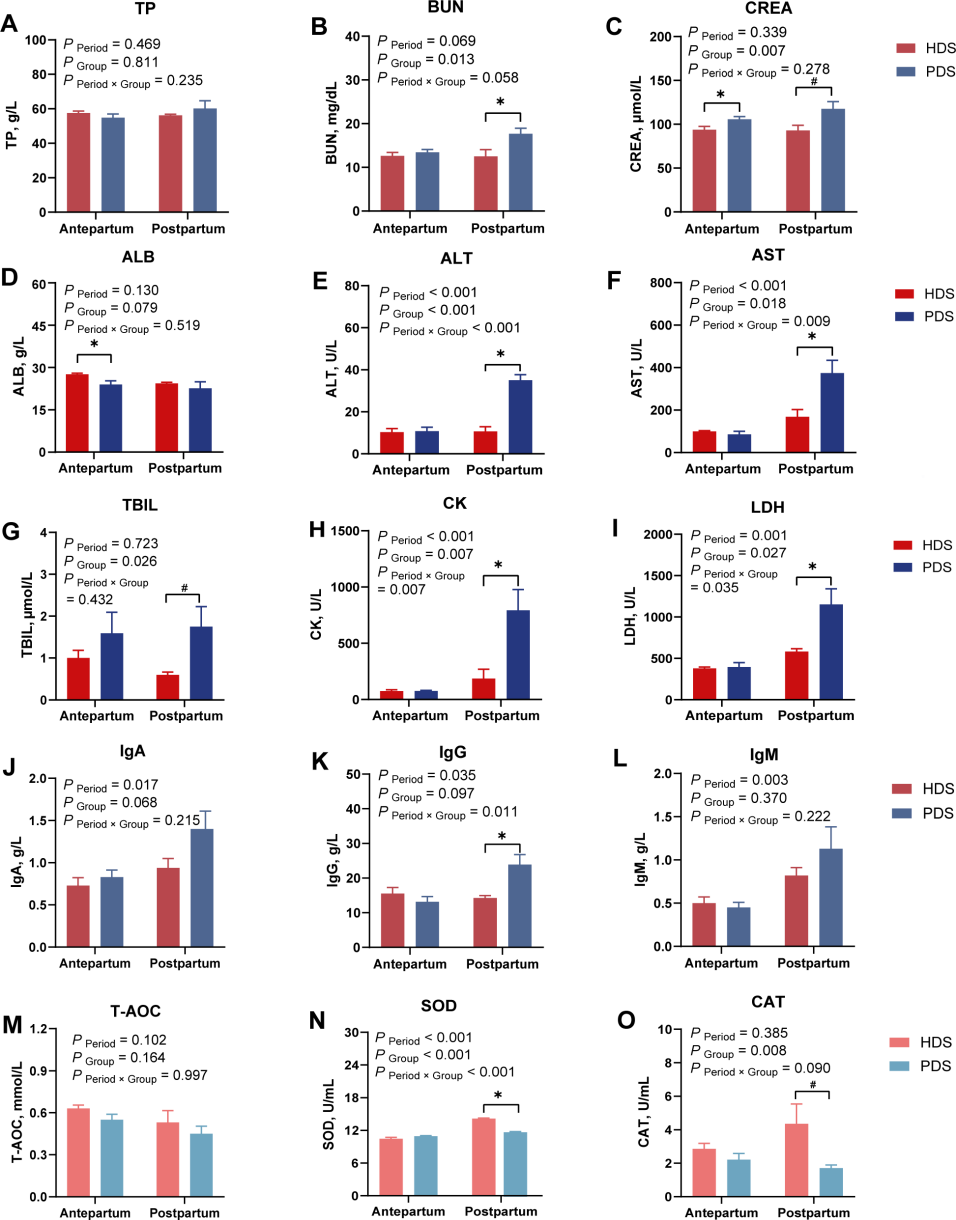
Plasma physiological parameters of healthy and paretic dairy sheep at different periods. (**A–C**) Protein metabolism of HDS and PDS groups in antepartum and postpartum. TP, total protein; BUN, blood urea nitrogen; CREA, creatinine. (**D–I**) Liver function of HDS and PDS groups in antepartum and postpartum. ALB, albumin; ALT, alanine aminotransferase; AST, aspartate aminotransferase; TBIL, total bilirubin, CK, creatine kinase; LDH, lactate dehydrogenase. (**J–L**) Systemic immunity index of HDS and PDS groups in antepartum and postpartum. IgA, immunoglobulin A; IgG, immunoglobulin G; IgM, immunoglobulin M. (**M–O**) Oxidative stress status of HDS and PDS groups in antepartum and postpartum. T-AOC, total antioxidant capacity; SOD, superoxide dismutase; CAT, catalase; HDS, healthy dairy sheep; PDS, paretic dairy sheep. *, significantly different between the two groups (*P* < 0.05). #, a tendency to differ between the two groups (0.05 < *P* < 0.10).

Regarding glucolipid metabolism, GLU, TC, HDL, and LDL levels were lower in PDS compared to HDS during antepartum (*P* < 0.05; Fig. 1F through I), whereas ChE showed a tendency to be higher (*P* = 0.085; Fig. 1K). Postpartum, TG levels in PDS were higher than those in HDS (*P* < 0.05; Fig. 1J). For protein metabolism, no difference was observed in plasma TP levels between HDS and PDS during antepartum and postpartum (*P* > 0.10; Fig. 2A). Antepartum, CREA levels in PDS were higher than those in HDS (*P* < 0.05; Fig. 2C). Postpartum, BUN levels in PDS were higher than those in HDS (*P* < 0.05; Fig. 2B), while CREA showed a tendency to be higher (*P* = 0.084; Fig. 2C). Regarding liver function, no difference was observed in ALP levels between HDS and PDS during antepartum and postpartum (*P* > 0.10; Fig. S1). Antepartum, ALB levels in HDS were higher than those in PDS (*P* < 0.05; Fig. 2D). Postpartum, ALT, AST, CK, and LDH levels in PDS were higher than those in HDS (*P* < 0.05; Fig. 2), while TBIL showed a tendency to be higher (*P* = 0.095; Fig. 2G).

### Immunity index and oxidative stress status

Regarding systemic immunity, no differences were observed in IgA and IgM levels between HDS and PDS (*P* > 0.10; Fig. 2J and L). Postpartum, IgG levels in PDS were higher than those in HDS (*P* < 0.05; Fig. 2K). Regarding oxidative stress, no differences were observed in T-AOC, GSH-Px, and MDA levels between HDS and PDS (*P* > 0.10; Fig. 2; Fig. S2). Postpartum, SOD levels in HDS were higher than those in PDS (*P* < 0.05; Fig. 2N), while CAT levels showed a tendency to be higher (*P* = 0.089; Fig. 2O).

### Elemental contents in plasma and colostrum

**Fig 3:**
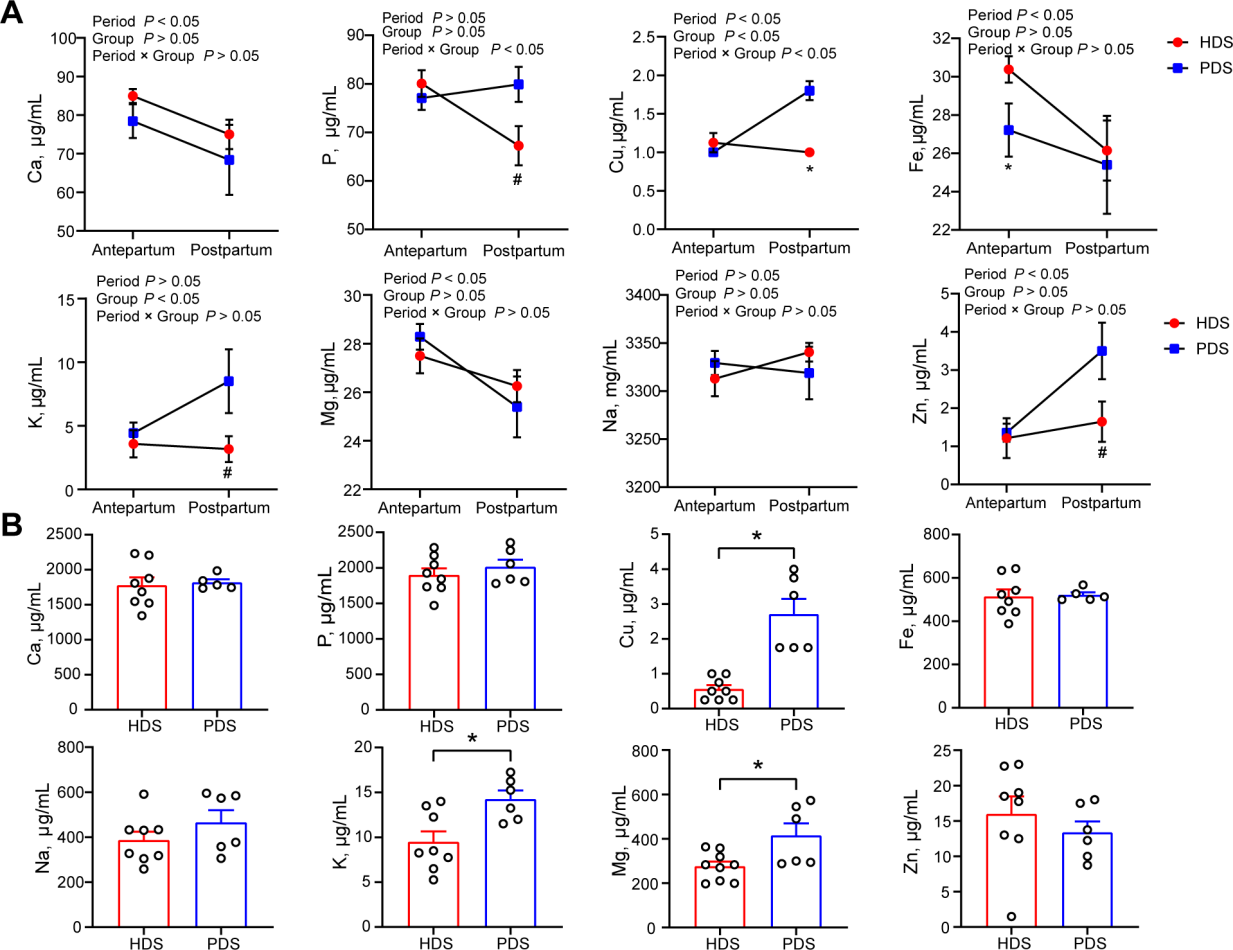
Contents of elements in plasma and colostrum of healthy and paretic dairy sheep at different periods. (**A**) Contents of elements in plasma of HDS and PDS groups in antepartum and postpartum. (**B**) Contents of elements in colostrum of HDS and PDS groups in antepartum and postpartum. Ca, calcium; P, phosphorus; Cu, copper; Fe, iron; Na, sodium; K, potassium; Mg, magnesium; Zn, zinc; HDS, healthy dairy sheep; PDS, paretic dairy sheep. *, significantly different between the two groups (*P* < 0.05). #, a tendency to differ between the two groups (0.05 < *P* < 0.10).

Antepartum, plasma Fe levels in PDS were lower than those in HDS (*P* < 0.05; Fig. 3A). Postpartum, plasma Cu levels in PDS were higher than those in HDS (*P* < 0.05). Additionally, plasma levels of P, K, and Zn in PDS showed an increasing trend compared to HDS (0.05 < *P* < 0.1). Colostrum Cu, K, and Mg levels in PDS were higher than those in HDS (*P* < 0.05; Fig. 3B).

### Identification of early biomarkers of paresis in dairy sheep

**Fig 4:**
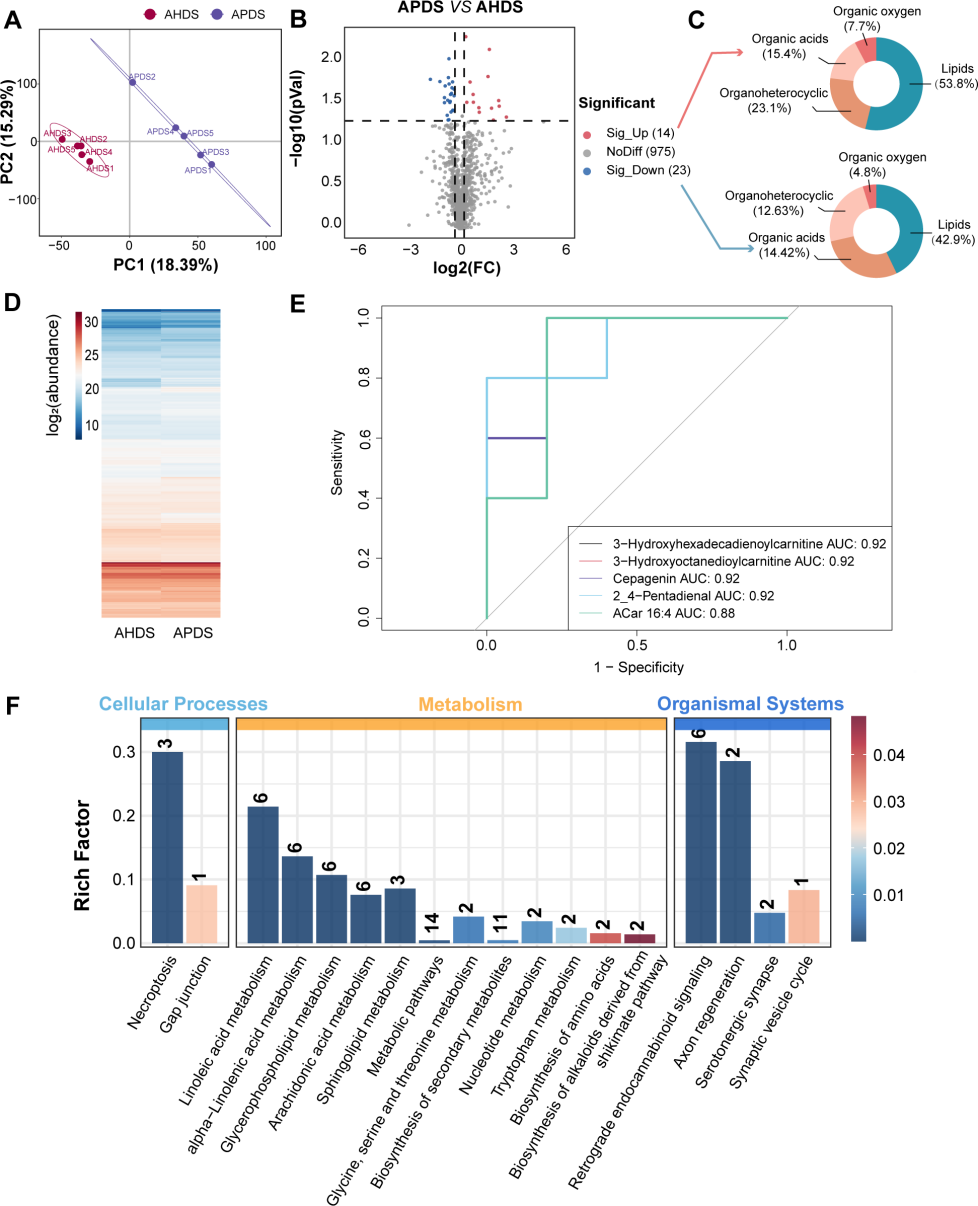
Metabolic characteristics in antepartum between healthy and paretic sheep. (**A**) Partial least squares discriminant analysis (PLS-DA) score chart between APDS and AHDS groups. (**B**) Volcano maps of differential metabolites between APDS and AHDS groups. (**C**) Differential metabolite HMDB Super class classification map. (**D**) Cluster heat map of metabolites between APDS and AHDS groups. (**E**) Receiver-operator characteristic curve of the top five VIP plasma metabolites in dairy sheep at 21 days before parturition. (**F**) KEGG pathway analysis of metabolite differences between APDS and AHDS groups. AHDS, healthy dairy sheep in antepartum; APDS, paretic dairy sheep in antepartum.

Plasma metabolite profiles during the antepartum period were assessed using untargeted LC-MS/MS-based metabolomics in both positive and negative ion modes. A total of 1,150 metabolites were identified for further analysis after removing impurity peaks and duplicate ions in both ionization modes. At the same time, we used cluster heat maps to visualize the expression levels of the same metabolite in different groups (Fig. 4D). Partial least squares discriminant analysis (PLS-DA) indicated a clear separation of plasma metabolites between the APDS and AHDS groups (Fig. 4A). Validation of the PLS-DA model was performed through 200 permutation tests (intercept of R2 = 0.9926, intercept of Q2 = −0.4231), ensuring that overfitting did not occur (Fig. S3). Subsequently, 37 differential metabolites were identified based on VIP > 1 and *P* < 0.05, with 14 metabolites upregulated and 23 metabolites downregulated in the APDS group compared to the AHDS group (Fig. 4B; Table S4). Categorization of the 37 differential metabolites through HMDB compound analysis revealed their classification into four classes: lipids and lipid-like molecules, organoheterocyclic compounds, organic acids and derivatives, and organic oxygen compounds. Among the upregulated or downregulated metabolites, lipids and lipid-like molecules are the predominant categories, constituting 53.8% and 42.9% of the total compound classification, respectively (Fig. 4C). Moreover, KEGG pathway enrichment analysis demonstrated that lipid metabolism is the main enrichment pathway, including linoleic acid metabolism, glycerophospholipid metabolism, and arachidonic acid metabolism (Fig. 4F; Table S5). An ROC plot showing the performance of the top five VIP metabolites (3-hydroxyhexadecadienoylcarnitine, 3-hydroxyoctanedioylcarnitine, cepagenin, 2,4-pentadienal, and ACar 16:4) in predicting which dairy sheep will develop paresis is shown in Fig. 4E. The AUC for this curve is 0.92 or 0.88, which indicates that these five biomarkers have very good predictive ability. We assessed the correlation between plasma metabolites and physiological parameters. Our analysis identified that physiological parameters such as GLU, TC, HDL, LDL, TG, and ALB were negatively associated with APDS-enriched metabolites (e.g., thymine and L-beta-homoleucine-HCl) or positively correlated with APDS-depleted metabolites (e.g., 2-acetylpyridine and L-tryptophan) (Fig. S4). Conversely, physiological parameters such as ChE, BUN, CREA, and SOD were positively associated with APDS-enriched metabolites.

### Microbial characteristics in postpartum between healthy and paretic sheep

**Fig 5:**
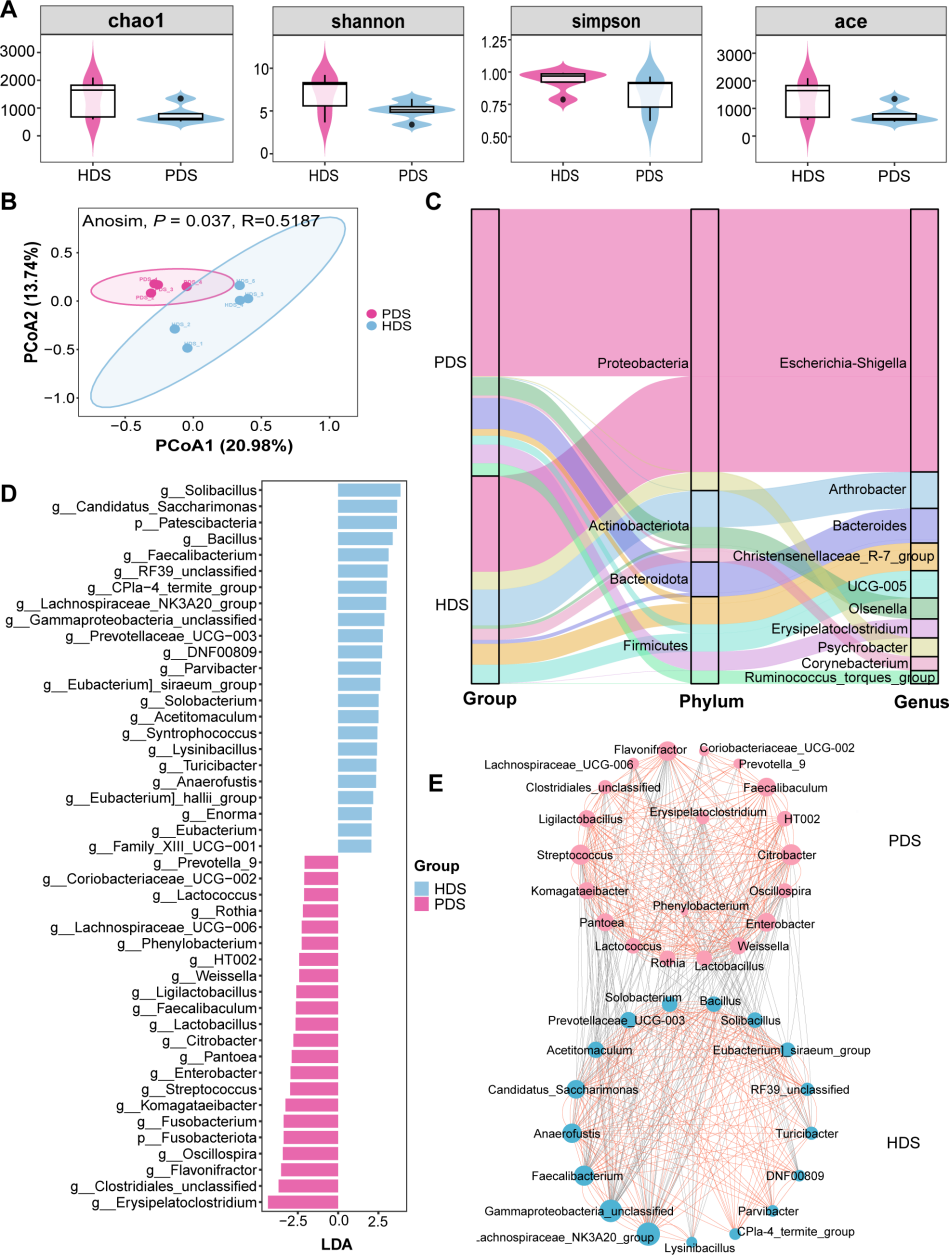
Microbial characteristics in postpartum between healthy and paretic sheep. (**A**) The alpha diversity parameters including Chao 1, Shannon, Simpson, and Ace index between PDS and HDS groups. (**B**) Beta diversity of principal co-ordinates analysis based on Jaccard dissimilarity matrices between PDS and HDS groups. (**C**) The relative abundance of phylum level and genus level (top 10) between PDS and HDS groups. (**D**) LEfSe bar plot of intestinal microbiota (LDA ≥ 2). (**E**) Network analysis to reveal differential microbial interactions between PDS and HDS groups. The network analysis showed the degree of correlation between the bacteria at the genus level (Spearman’s |*r*| > 0.7 and adjusted *P* < 0.05). Lines between two nodes represent the correlation, with a red line indicating a positive correlation and a gray line indicating a negative correlation. HDS, healthy dairy sheep in postpartum; PDS, paretic dairy sheep in postpartum.

Venn diagrams showed that the HDS and PDS groups shared 1,234 identical core ASVs (Fig. S5). Additionally, the HDS group exhibited 3,619 unique ASVs, whereas the PDS group contained 1,546 ASVs (Fig. S5). Alpha diversity calculations revealed no marked divergence of the Chao 1, Shannon, Simpson, and Ace index, indicating unchanged bacterial richness and evenness between the PDS and HDS (*P* > 0.05) (Fig. 5A). We employed the PLS-DA method to discriminate between different categories and capture potential differences in microbial community structure and composition more effectively. PLS-DA demonstrated disparity in microbial structure and composition between the HDS and PDS group (Fig. 5B). At the phylum level, the top four microorganisms in both groups were *Proteobacteria*, *Firmicutes*, *Actinobacteriota,* and *Bacteroidota*, which collectively accounted for over 94% of all identified phyla (Fig. 5C). At the genus level, the top five microorganisms in both groups were *Escherichia-Shigella*, *Arthrobacter*, *Bacteroides*, *Christensenellaceae_R-7_group,* and *UCG-005. Escherichia-Shigella* was the most abundant genus, accounting for 39.59% and 22.67% in the HDS and PDS groups, respectively (Fig. 5C). Furthermore, LEfSe analysis revealed that *Fusobacteriota* at the phylum level and *Erysipelatoclostridium*, *Flavonifractor*, *Oscillospira*, *Fusobacterium, Komagataeibacter*, and other genera at the genus level were enriched in the PDS group compared to the HDS group (Fig. 5D). Conversely, *Patescibacteria* at the phylum level, *Solibacillus*, *Bacillus*, *Faecalibacterium, Candidatus_Saccharimonas*, and other genera at the genus level were enriched in the HDS group compared to the PDS group. We further analyzed the correlations among differential microbiota (Fig. 5E). The identified microorganisms formed two distinct networks, predominantly enriched in the PDS and HDS groups, respectively. A negative correlation was observed between the microbiota of the PDS and HDS groups, whereas a positive correlation was evident within each group. Metabolic functions of the intestinal microbiota were predicted utilizing PICRUSt2 based on the KEGG database (Table S6). In the PDS group, lipid metabolism and xenobiotics biodegradation decreased, while signaling molecules and interactions increased compared to the HDS group (Table S6).

### Metabolic characteristics in postpartum between healthy and paretic sheep

**Fig 6:**
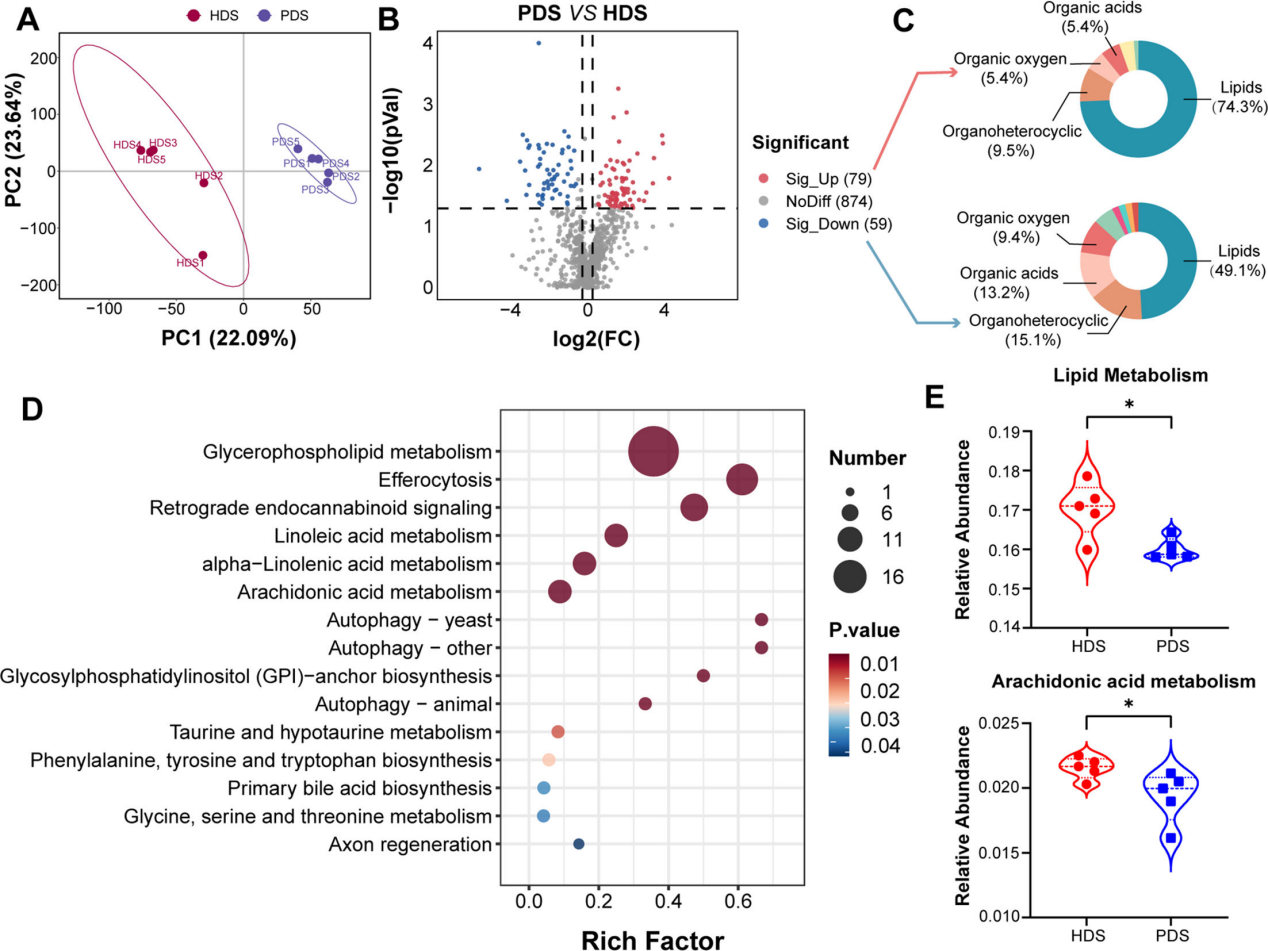
Comparison of metabolite characteristics postpartum between healthy and paretic sheep. (**A**) PLS-DA score chart between PDS and HDS groups. (**B**) Volcano maps of differential metabolites between PDS and HDS groups. (**C**) Differential metabolite HMDB Super class classification map. (**D**) KEGG pathway analysis of metabolite differences between PDS and HDS groups. (**E**) PICRUSt2 predicts the relative abundance of lipid metabolism and arachidonic acid metabolism in fecal microbes between PDS and HDS groups. AHDS, healthy dairy sheep in antepartum; APDS, paretic dairy sheep in antepartum; HDS, healthy dairy sheep in postpartum; PDS, paretic dairy sheep in postpartum.

PLS-DA revealed a separation of plasma metabolites between PDS and HDS (Fig. 6A). The results of the permutation test (intercept of R2 = 0.935, intercept of Q2 = −0.707) showed that the PLS-DA model was not over-fitted (Fig. S6). Subsequently, 138 differential metabolites were identified, with 79 metabolites upregulated and 59 metabolites downregulated in the PDS group compared to the HDS group (Fig. 6B; Table S7). Among the upregulated or downregulated metabolites, lipids and lipid-like molecules are the predominant categories, constituting 74.3% and 49.1% of the total compound classification, respectively (Fig. 6C). Moreover, KEGG pathway enrichment analysis demonstrated that lipid metabolism is the main enrichment pathway, such as glycerophospholipid metabolism, linoleic acid metabolism, alpha-linolenic acid metabolism, and arachidonic acid metabolism (Fig. 6D; Table S8). Interestingly, the PICRUSt2 analysis of intestinal flora metabolic functions revealed that the relative abundances of lipid metabolism and arachidonic acid metabolism were higher in the HDS group compared to the PDS group (Fig. 6E). Among the differential metabolites identified in antepartum and postpartum, seven metabolites showed consistent expression patterns, including two downregulated metabolites (L-tryptophan and D-tryptophan) and five upregulated metabolites (2,4-pentadienal, cis-4-decenoylcarnitine, 3-hydroxyoctanedioylcarnitine, 3-hydroxyisovalerylcarnitine, and hexadecanedioic acid-mono-L-carnitine ester) (Fig. S7A). These metabolites were strongly correlated with those enriched in lipid metabolism pathways during both antepartum and postpartum periods (Fig. S7B and C).

### Integration analysis of the differential microbes, function, and metabolites

**Fig 7:**
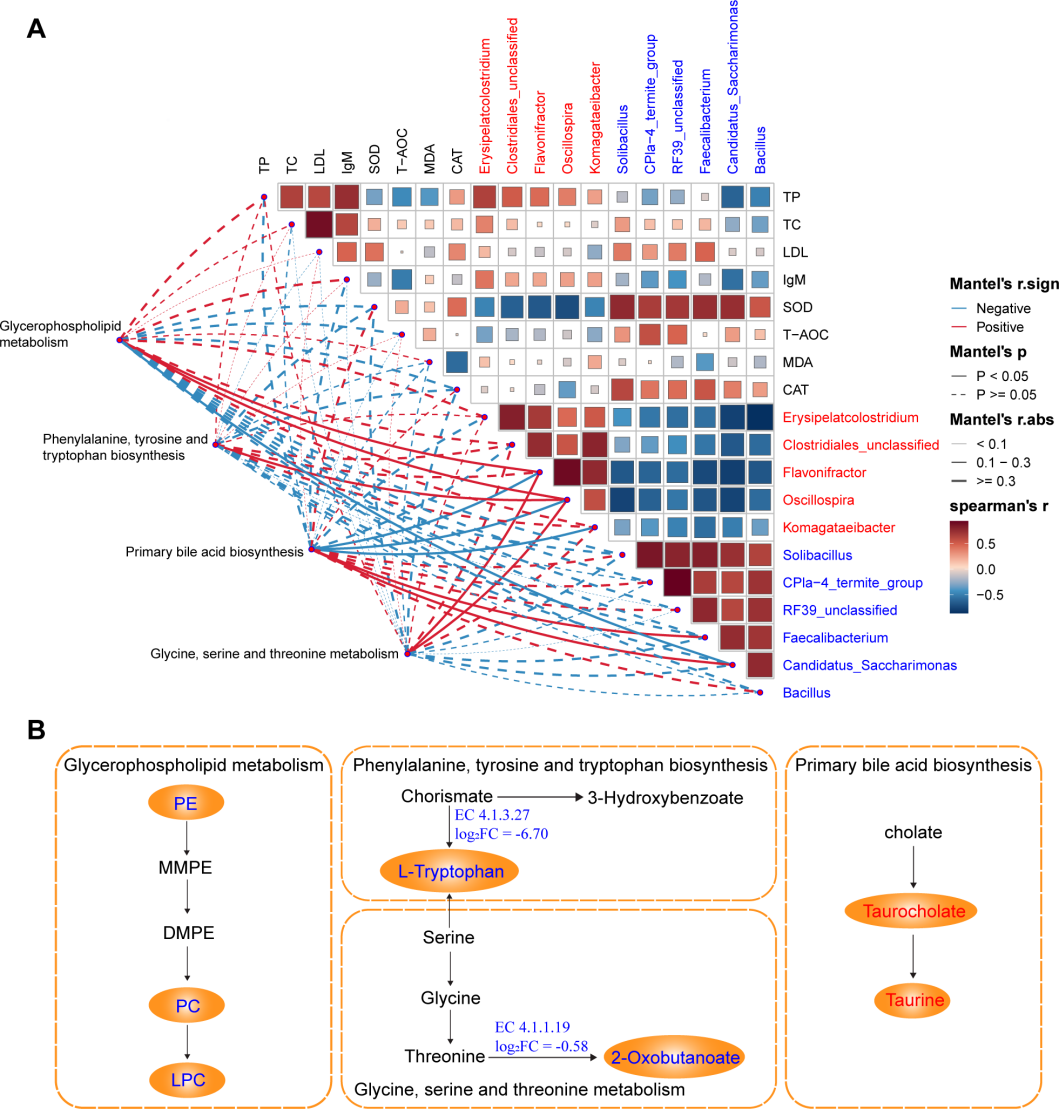
The integration analysis of the significantly differential microbes, function, and metabolites. (**A**) The Spearman correlations among the significantly differential microbiota, physiological parameters, and the enriched metabolic pathways. (**B**) Integration of significantly different metabolic pathways. Red and blue words represent what was significantly increased and decreased in PDS compared with HDS, respectively, and black words indicate no significant difference observed between the two groups. EC 4.1.3.27 represents anthranilate synthase. EC 4.1.1.19 represents arginine decarboxylase. PE, phosphatidylethanolamine; MMPE, monomethyl-phosphatidyl-ethanolamine; DMPE, dimethyl-phosphatidyl-ethanolamine; PC, phosphatidylcholine; LPC, lysophosphatidylcholine; HDS, healthy dairy sheep in postpartum; PDS, paretic dairy sheep in postpartum.

The correlation analysis showed that the differential genera were strongly correlated with these pathways (Fig. 7A). Glycerophospholipid metabolism exhibited positive correlations with *Flavonifractor* and *Oscillospira*, but was negatively correlated with *Candidatus_Saccharimonas*. Phenylalanine, tyrosine, and tryptophan biosynthesis showed a positive correlation with *Oscillospira*. Primary bile acid biosynthesis was negatively correlated with *Flavonifractor*, *Oscillospira*, and *Komagataeibacter*, but positively correlated with *Faecalibacterium* and *Candidatus_Saccharimonas*. Glycine, serine, and threonine metabolism showed positive correlations with *Flavonifractor* and *Oscillospira*. Additionally, the metabolites and key enzymes involved in the identified KEGG pathways showed differences between the PDS and HDS groups (Fig. 7B). The enzymes EC 4.1.3.27 and EC 4.1.1.19 were more abundant in HDS than those in the PDS group (*P* < 0.05). Integration of changes between the two groups, including metabolite abundance and associated metabolic pathways, revealed that lysophosphatidylcholine (LPC) synthesis in glycerophospholipid metabolism, L-tryptophan synthesis in phenylalanine, tyrosine, and tryptophan biosynthesis, and 2-oxobutanoate synthesis in glycine, serine, and threonine metabolism were lower in the PDS group than in the HDS group. However, taurine synthesis in primary bile acid biosynthesis was higher in the PDS group (Fig. 7B).

### Alteration of plasma metabolites in stages of paresis progression

**Fig 8:**
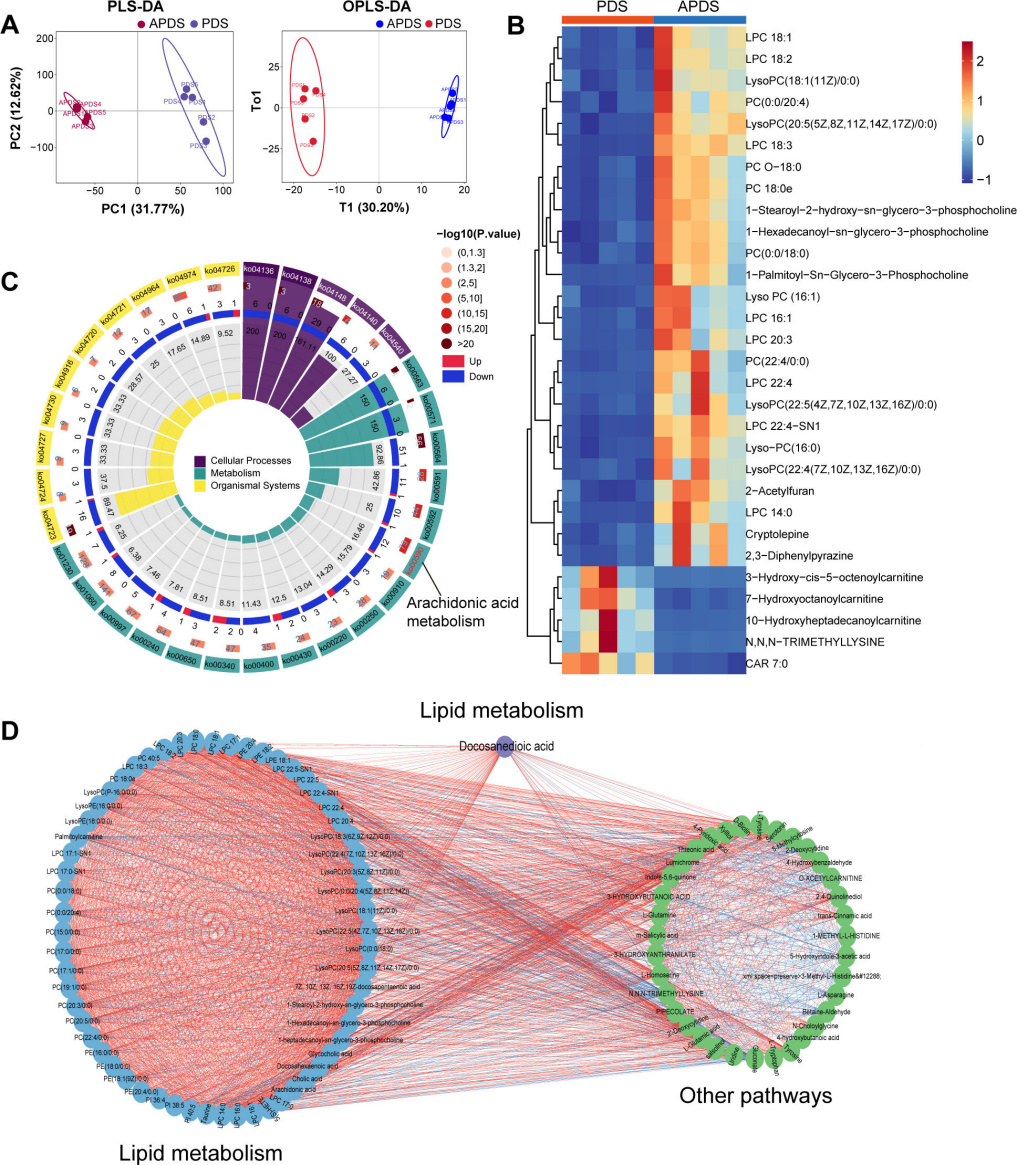
Metabolic characteristics in antepartum between PDS and APDS groups. (**A**) Partial least squares discriminant analysis (PLS-DA) and orthogonal partial least squares discriminant analysis (OPLS-DA) score chart between PDS and APDS groups. (**B**) Cluster heat map of differential metabolites of top 30. (**C**) The enrichment circle diagram of differential metabolites was based on KEGG function analysis. (**D**) Correlation network in plasma metabolites between PDS and APDS groups. APDS, paretic dairy sheep in antepartum; PDS, paretic dairy sheep in postpartum.

PLS-DA and OPLS-DA analyses revealed differences in the plasma metabolome between the APDS and PDS groups (Fig. 8A; Fig. S8 and S9). To characterize altered plasma metabolites in stages of paresis progression, we identified 282 differential metabolites, with 141 metabolites upregulated and 141 metabolites downregulated in the APDS group compared to the PDS group (Table S9). Among the top 30 differential metabolites, those with higher relative abundance in the PDS group compared to the APDS group included 3-hydroxy-cis-5-octenoylcarnitine, 7-hydroxyoctanoylcarnitine, 10-hydroxyheptadecanoylcarnitine, CAR 7:0, and N,N,N-trimethyllysine. In contrast, the relative abundance of LPC 18:1, LPC 18:2, LysoPC (18:1(11Z)/0:0), PC (0:0/20:4), and others in the PDS group was lower than in the APDS group (Fig. 8B). We primarily focused on the pathways of differential metabolites enriched in cellular processes, metabolism, and organismal systems (Fig. 8C; Table S10). The top three differential metabolic pathways in the PDS group compared to the APDS group were glycosylphosphatidylinositol-anchor biosynthesis, lipoarabinomannan biosynthesis, and glycerophospholipid metabolism. Additionally, differential metabolites were enriched in the arachidonic acid metabolism pathway between the PDS and APDS groups. The correlation network of altered metabolites and metabolic pathways was evaluated for the PDS and APDS groups. Our analysis showed that differential metabolites in plasma were mostly associated with lipid metabolism, of which these lipid metabolomes formed a separate cluster from other metabolites and metabolic pathways (Fig. 8D). Furthermore, docosanedioic acid (DDA) in lipid metabolism was located at the center of correlation network, thus suggesting its significance in plasma during stages of paresis progression.

## DISCUSSION

Our study observed changes in glucolipid metabolism, protein metabolism, liver function, immunity, oxidative stress, and plasma element indices in dairy sheep with paresis. Furthermore, potential early diagnostic biomarkers, including acylcarnitines, were identified by comparing plasma metabolic profiles between the APDS and AHDS groups (both pre-disease). Integration of multi-omics data revealed that lipid and amino acid metabolism changes are closely linked to paresis. Additionally, DDA was found to play a pivotal role in the progression of paresis. Ultimately, this study offers valuable insights for devising strategies to prevent and manage periparturient metabolic disorders in dairy sheep.

East Friesian-Hu crossbred sheep, recognized for their genetic potential to produce multiple offspring, experience high energy demands during pregnancy to support fetal development (24). During late gestation, rapid fetal growth compresses abdominal organs, limiting maternal feed intake. This causes an energy deficit, where expenditure surpasses intake, leading to NEB in the ewes (32). Plasma GLU levels are strongly correlated with NEB status in ruminants during the transition period (33). Ma et al. (34) monitored the energy balance of 153 Holstein-Friesian cows during weeks 1–6 in lactation and found that cows experiencing severe NEB had lower plasma GLU levels (2.96 vs 3.57 mmol/L) compared to those with stable positive energy balance. In our study, prior to the onset of clinical symptoms, APDS exhibited lower GLU levels (2.39 vs 3.34 mmol/L) than AHDS, suggesting that APDS are more prone to energy imbalance in the early period. Consistently, Xue et al. (35) reported lower blood GLU levels in a pregnancy toxemia model induced by 70% feed restriction in ewes. Furthermore, our study demonstrated reductions in TC, HDL, and LDL in APDS compared to AHDS (1.19 vs 0.99 mmol/L, 0.67 vs 0.56 mmol/L, and 0.69 vs 0.54 mmol/L, respectively). This finding aligns with the cholesterol metabolic state observed in dairy cows during the transition period by Kessler et al. (36). To maximize energy supply, ewes mobilize body energy reserves through adaptive mechanisms, leading to fat mobilization. Adipose tissue is catabolized into non-esterified fatty acids (NEFAs) and glycerol (12). In the liver, the glycerol may be used to produce GLU or be recombined with NEFAs to make TG (12). However, excessive NEFA accumulation surpasses hepatic oxidative capacity and very-low-density lipoprotein (VLDL) synthesis rates, leading to intracellular TG accumulation in hepatocytes. However, no significant differences in plasma TG levels were observed in our study between APDS and AHDS, potentially due to the limited hepatic export of TG that can be transported out of the liver by VLDL (37). Additionally, APDS exhibited increased CREA levels (93.78 vs 105.71 µmol/L) and decreased ALB levels (27.84 vs 24.17 g/L) compared to AHDS, indicating accelerated muscle catabolism and impaired hepatic synthetic function. Early declines in plasma GLU, TC, and ALB, accompanied by elevated CREA levels, may serve as potential biomarkers for predicting paresis; however, large-scale validation studies are warranted.

Following the onset of paresis, TG levels in PDS were elevated compared to HDS (0.09 vs 0.27 mmol/L). This observation is consistent with the findings of Zhou et al. (38), who reported that subclinical ketosis cows suppress cholesterol synthesis via the downregulation of acetyl-coenzyme A acetyltransferase 2 while simultaneously increasing TG synthesis, ultimately resulting in disrupted TG and cholesterol metabolism. Moreover, in our study, PDS exhibited elevated levels of ALT (10.72 vs 35.05 U/L), AST (168.33 vs 374.35 U/L), CK (187.29 vs 792.78 U/L), and LDH (582.71 vs 1,152.57 U/L) compared to HDS, indicating hepatic dysfunction (39, 40). Additionally, the observed decline in SOD levels (14.17 vs 11.83 U/L) in PDS suggests impaired free radical scavenging capacity and reduced antioxidant defense. The activation of the immune system and the exacerbation of inflammatory responses are mediated by cytokine release (41). Zhao et al. (40) demonstrated that in ketotic cows, increased levels of serum pro-inflammatory cytokines, including transforming growth factor-beta, tumor necrosis factor-alpha, interferon-gamma, and interleukin-1 beta, indicate the presence of systemic chronic low-grade inflammation. Consistently, in our study, elevated IgG levels (14.29 vs 23.89 g/L) in PDS may reflect an amplified inflammatory response to pathological damage linked to paresis (42). This immune activation may represent an adaptive mechanism aimed at mitigating disease progression.

In this study, no differences in plasma Ca and P levels were observed between PDS and HDS. These findings indicate that Ca and P metabolism dysregulation is unlikely a primary feature of periparturient paresis, likely due to tightly regulated Ca-P homeostasis in dairy sheep (43). Hubner et al. (44) showed that the serum Ca (2.36 vs 2.22 mmol/L) and Mg (0.89 vs 0.81 mmol/L) of primiparous and multiparous cows with subclinical hyperketonemia were lower than those of normal cows. Nonetheless, differences in other trace elements were observed in our study, possibly reflecting the influence of immune responses or metabolic disturbances caused by paresis on mineral metabolism. The increase in Cu levels is usually mediated by the synthesis and release of ceruloplasmin in the liver, which is particularly evident in response to microbial invasion or inflammatory stimuli (45). Additionally, lower Fe levels were observed in APDS, which may be relaated to the “hepcidin-mediated Fe regulation mechanism” in the context of chronic disease or inflammation. Elevated hepcidin levels during chronic inflammation inhibit Fe release from cells into the bloodstream, resulting in reduced serum Fe levels (46). Analysis of colostrum mineral composition revealed higher levels of Cu, Fe, and K in the PDS group compared to the healthy group. This mineral accumulation may be associated with impaired renal function in paretic dairy sheep. Renal dysfunction reduces mineral excretion, causing their accumulation in milk (47). Increased plasma CREA (92.99 vs 117.66 µmol/L) and BUN (12.53 vs 17.67 mg/dL) levels in our study indicate kidney damage.

Acylcarnitines are products of fatty acid metabolism, formed by the esterification of fatty acids with L-carnitine. The primary biological function of acylcarnitines is to transport acyl groups from the cytoplasm to the mitochondrial matrix for β-oxidation, generating energy to sustain cellular activity (48, 49). The concentration and composition of acylcarnitines reflect the status of fatty acid metabolism, particularly the balance between fatty acid oxidation, energy supply, and storage. Under conditions of high energy demand, such as exercise or fasting, fatty acid oxidation and acylcarnitine production increase to meet the energy requirements (50). During the transition period, the oxidation of fatty acids in dairy cows may be incomplete, leading to the accumulation of acylcarnitines, which could be due to insufficient metabolic adaptation to cope with the metabolic load of fatty acids (51). Our research demonstrates that acylcarnitines (3-hydroxyhexadecadienoylcarnitine and 3-hydroxyoctanedioylcarnitine) exhibit a high degree of predictive power for paresis. Several recent metabolomics studies have revealed that carnitines and acylcarnitines can be used as potential diagnostic biomarkers in overconditioned cows (52, 53). Zhao et al. (54) demonstrated that, compared to cows with low lipolysis, cows with high lipolysis had higher serum concentrations of acylcarnitines in the early postpartum period, consistent with their greater degree of lipolysis and state of energy deficit. Moreover, acylcarnitine changes correlated with metabolic parameters, including insulin sensitivity, liver function, oxidative stress, and inflammatory responses. These findings highlight their significant role in metabolic adaptation during the periparturient period and potential links to metabolic disorders. Huang et al. (55) showed that dairy goats with subclinical hyperketonemia had higher plasma concentrations of certain acylcarnitines (such as isovalerylcarnitine, O-adipoylcarnitine, L-acetylcarnitine) compared to healthy goats, suggesting that these goats may experience a more severe energy deficit, leading to increased lipolysis and consequently affecting the metabolism of acylcarnitines. In summary, acylcarnitines may serve as potential biomarkers for assessing the metabolic status during the transition period, aiding in the early identification and management of metabolic diseases, such as paresis.

Interestingly, seven metabolites exhibit similar expression patterns in the APDS vs AHDS, PDS vs HDS, and PDS vs APDS comparisons. These seven differential metabolites include L-tryptophan, D-tryptophan, 3-hydroxyoctanedioylcarnitine, 3-hydroxyisovalerylcarnitine, 2,4-pentadienal, cis-4-decenoylcarnitine, and hexadecanedioic acid-mono-L-carnitine ester. The metabolites of tryptophan are not only closely associated with neurotransmitter synthesis but also interact intricately with multiple physiological processes, including systemic energy metabolism, immune responses, oxidative stress, and the regulation of intestinal homeostasis (56). With the advancing understanding of the tryptophan metabolic pathway, this amino acid has demonstrated potential as a biomarker in the diagnosis, prognostic assessment, and therapeutic monitoring of numerous diseases (56, 57). Our study reveals that DDA plays a critical role in the progression of paresis. DDA is also involved in the composition of cell membranes and is an important component of the antioxidant defense system (58, 59). The functional ramifications of these disparate metabolites, along with their correlation to periparturient paresis, merit further investigation.

The gut microbiota of healthy dairy sheep and those with periparturient paresis exhibit distinct compositional differences that may impact host metabolism and health through various pathways. In PDS, the enrichment of genera such as *Erysipelatoclostridium* and *Clostridiales_unclassified* indicates potential disruptions in immune barriers and metabolic balance. *Erysipelatoclostridium*, known for translocating across intestinal barriers and producing inflammatory toxins, may exacerbate systemic inflammation, as seen in inflammatory bowel disease (60). The increased abundance of *Flavonifractor* and *Oscillospira* in PDS highlights their roles in oxidative stress, low-grade inflammation, and glucose-lipid metabolic disorders, conditions commonly associated with diabetes and other chronic diseases (61–63). Furthermore, the presence of *Fusobacterium*, a genus that disrupts the intestinal barrier via adhesins and endotoxins, emphasizes its role in exacerbating systemic and intestinal inflammation (64). In contrast, the reduction of beneficial taxa such as *Candidatus_Saccharimonas* and *Faecalibacterium* may weaken the gut’s protective functions. *Candidatus_Saccharimonas* contributes to short-chain fatty acid production (65), which is crucial for intestinal barrier integrity and energy metabolism. Meanwhile, *Faecalibacterium*, recognized for its anti-inflammatory properties, protects against intestinal inflammation (66), highlighting the loss of microbiota-associated protective effects in PDS. These findings emphasize the critical role of gut microbiota alterations in the pathophysiology of periparturient paresis and underscore the need for further research to explore the mechanisms linking gut microbiota and host health in this condition.

The integrated analysis of microbial communities, functional pathways, and metabolites reveals that disorder in lipid metabolism and amino acid metabolism pathways may underlie the mechanisms of periparturient paresis in dairy sheep. Notably, the marked reduction in phosphatidylcholine (PC), phosphatidylethanolamine, and LPC suggests impaired glycerophospholipid metabolism. These disruptions may compromise the structural integrity and functionality of cell membranes, thereby affecting neurotransmitter release and signal transduction (67). Furthermore, disruptions in glycerophospholipid metabolism may worsen energy imbalances by interfering with the tricarboxylic acid cycle, ultimately impairing energy supply (68). Regarding amino acid metabolism, reduced levels of tryptophan and 2-oxobutyric acid may hinder the glutathione synthesis pathway, lowering antioxidative capacity, exacerbating oxidative stress, and ultimately causing neuronal damage and dysfunction. Alterations in bile acid metabolism also deserve attention (69, 70). Elevated levels of taurine and taurocholic acid in the primary bile acid biosynthesis pathway may indicate a compensatory response to neural damage. These metabolites are known to play critical roles in bile acid conjugation, anti-inflammatory processes, and neuroprotection. Similar to our findings, disruptions in lipid and amino acid metabolism pathways have been observed in high-lipolysis cows and dairy goats with subclinical hyperketonemia (54, 55). In addition, the arachidonic acid metabolism pathway was consistently enriched among the differential metabolites identified in the APDS vs AHDS, PDS vs HDS, and PDS vs APDS comparisons, with consistent results from PICRUSt2 KEGG function prediction. Arachidonic acid metabolism primarily occurs through the cyclooxygenase and lipoxygenase pathways, generating bioactive molecules like prostaglandins and leukotrienes (71). Metabolites derived from arachidonic acid play essential roles in various physiological and pathological processes, including inflammation, immune regulation, thrombosis, neural modulation, and cancer progression (71, 72). In paretic dairy sheep, activation of the arachidonic acid metabolism pathway may regulate local and systemic inflammatory responses, impair neural function, and exacerbate disease progression.

### Conclusions

Physiological, metabolomic, and microbiota differences were observed between healthy and paretic dairy sheep during the periparturient period. Antepartum metabolomics identified 37 differential metabolites, such as acylcarnitines, as potential early diagnostic markers. Gut microbiota analysis revealed genera such as *Fusobacterium* and *Erysipelatoclostridium* enriched in paretic sheep, and *Faecalibacterium* and *Bacillus* in healthy individuals. Multi-omics integration highlighted lipid and amino acid metabolism as key pathways involved in paresis. These findings offer new insights for early diagnosis and nutritional prevention of periparturient metabolic disorders in dairy sheep.

## Data Availability

The sequencing data obtained in this study were deposited in the NCBI Sequence Read Archive (SRA), accession number: PRJNA1206429.

## SUPPLEMENTAL MATERIAL

Supplemental figures spectrum.01544-25-s0001.docx Figures S1 to S9.

Supplemental tables spectrum.01544-25-s0002.docx Tables S1 to S10.

ASM does not own the copyrights to Supplemental Material that may be linked to, or accessed through, an article. The authors have granted ASM a non-exclusive, world-wide license to publish the Supplemental Material files. Please contact the corresponding author directly for reuse.
