# Supplementary material for: Integrated multi-omics identifies dysregulated lipid metabolism of paresis in dairy sheep during the early transition period
Source: Microbiol Spectr. 2025 Oct 9;13(11):e01544-25. doi: 10.1128/spectrum.01544-25 (PMC12584643; doi:10.1128/spectrum.01544-25)
Supplement: Supplemental figures — Figures S1 to S9. [file spectrum.01544-25-s0001.docx]

**Additional figure**


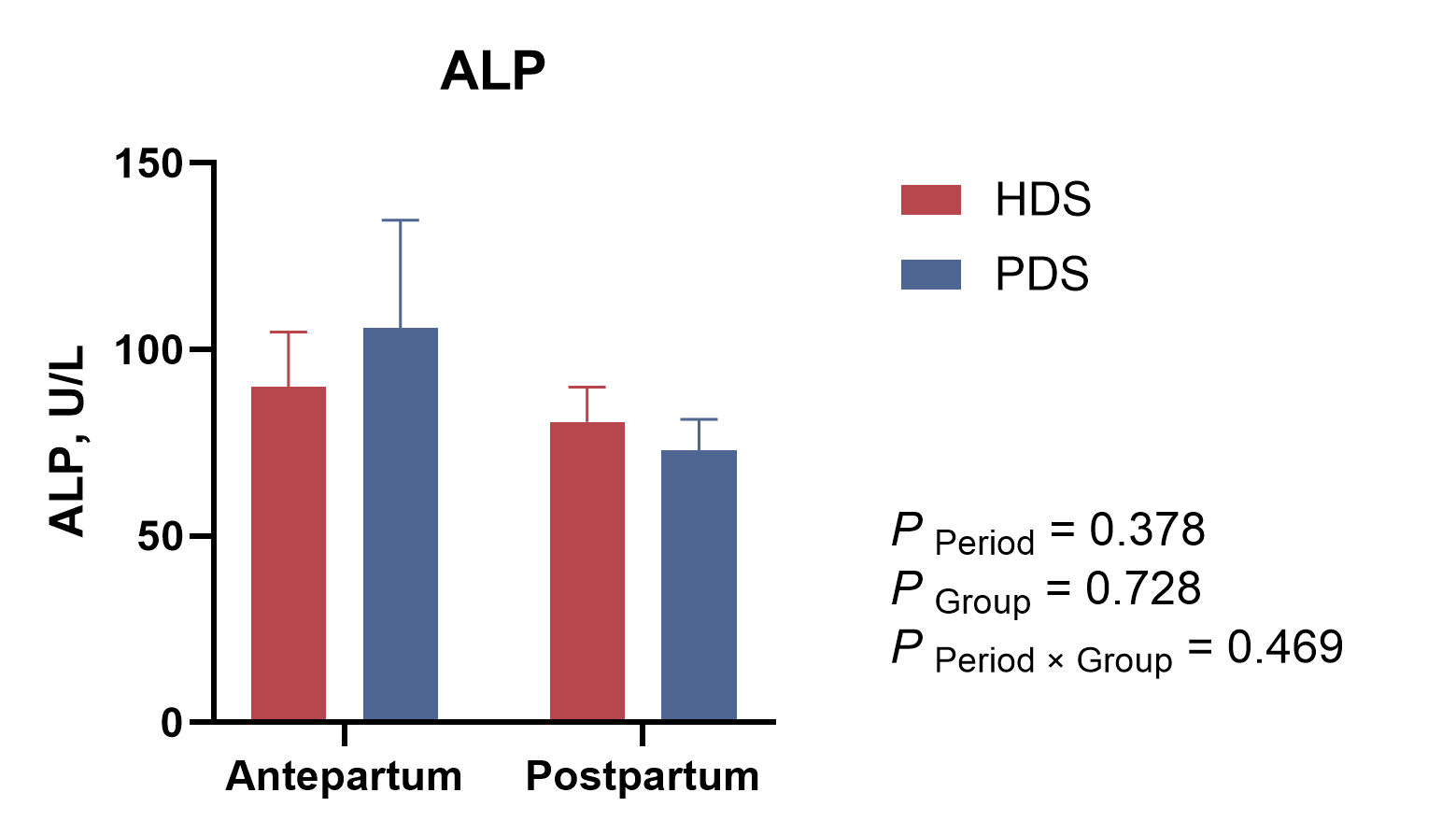


**Fig S1** The content of ALP in plasma between HDS and PDS groups in antepartum and postpartum. ALP, Alkaline phosphatase; HDS, healthy dairy sheep; PDS, paretic dairy sheep.


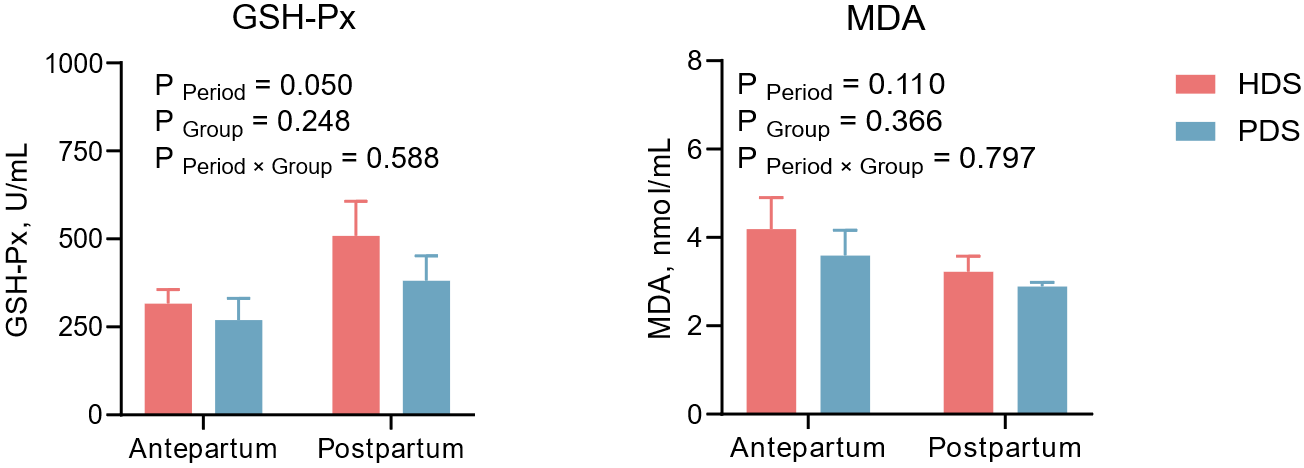


**Fig S2** The content of GSH-Px and MDA in plasma between HDS and PDS groups in antepartum and postpartum. GSH-Px, Glutathione peroxidase; MDA, Malondialdehyde; HDS, healthy dairy sheep; PDS, paretic dairy sheep.


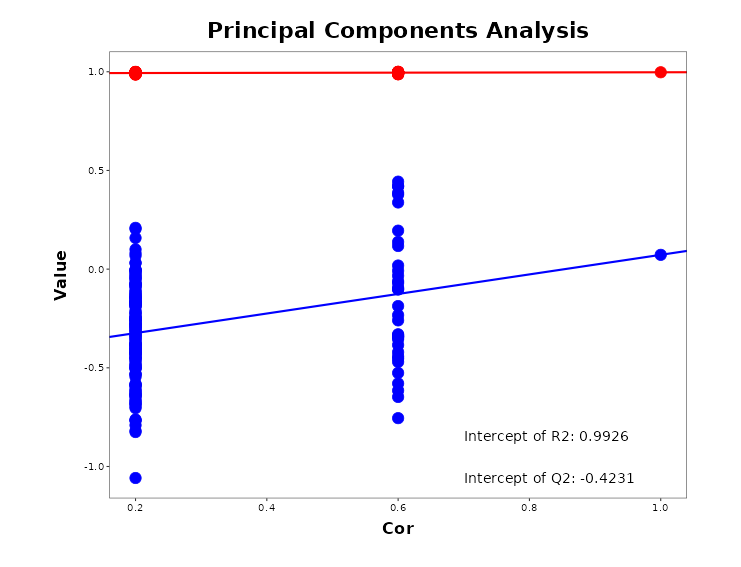


**Fig S3** Partial Least Squares Discriminant Analysis (PLS-DA) permutation testing plots between AHDS and APDS group. AHDS, healthy dairy sheep in antepartum; APDS, paretic dairy sheep in antepartum.


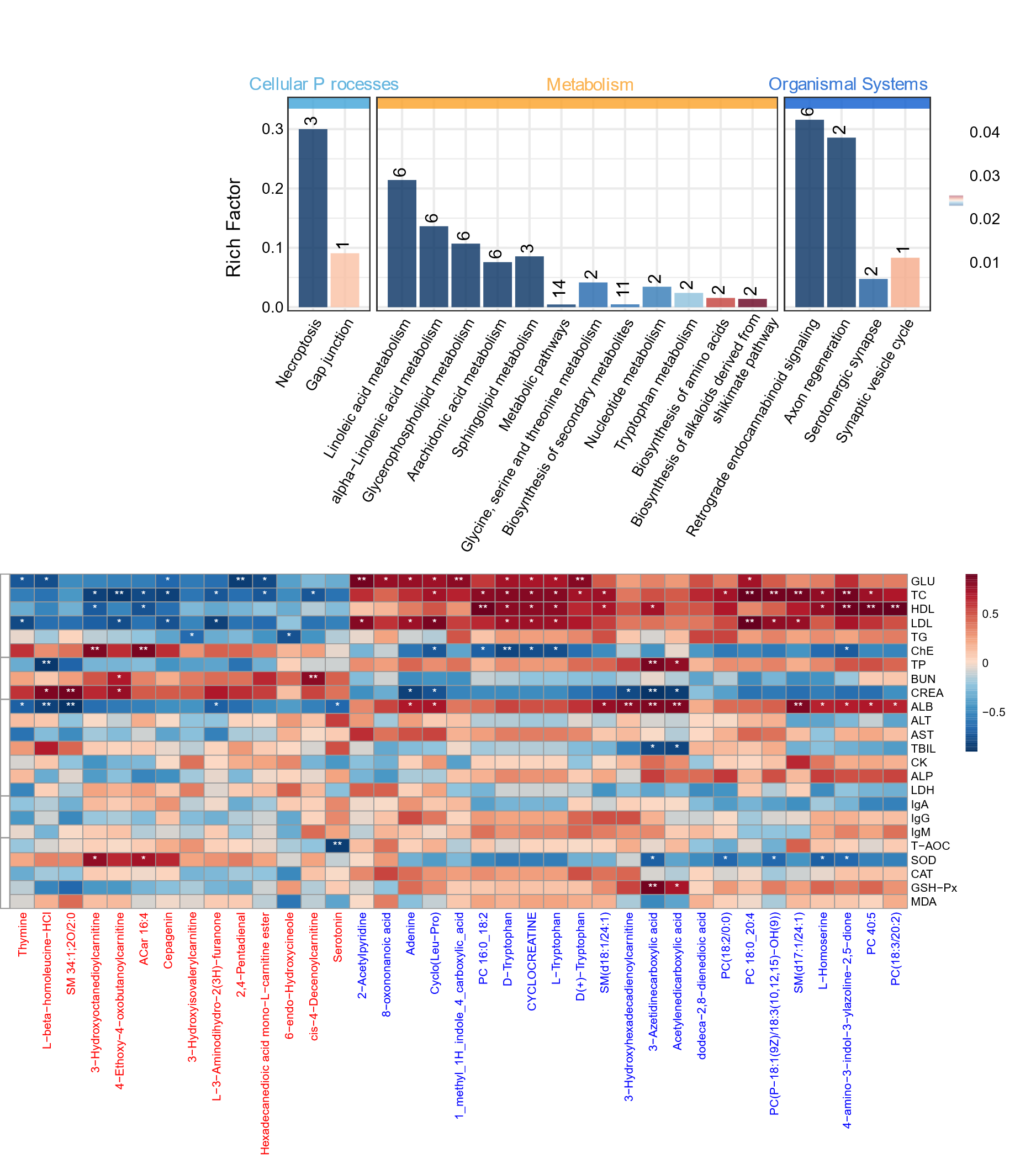


**Fig S4** Spearman correlation analysis between differential plasma metabolites and physiological parameters of APDS and AHDS groups. * *P* < 0.05, ** *P* < 0.01.


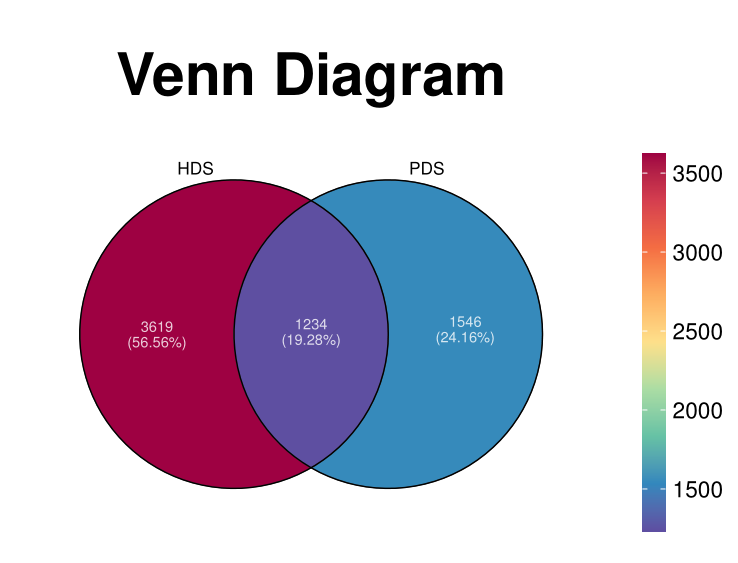


**Fig S5** Venn diagram of ASVs level. HDS, healthy dairy sheep in postpartum; PDS, paretic dairy sheep in postpartum.


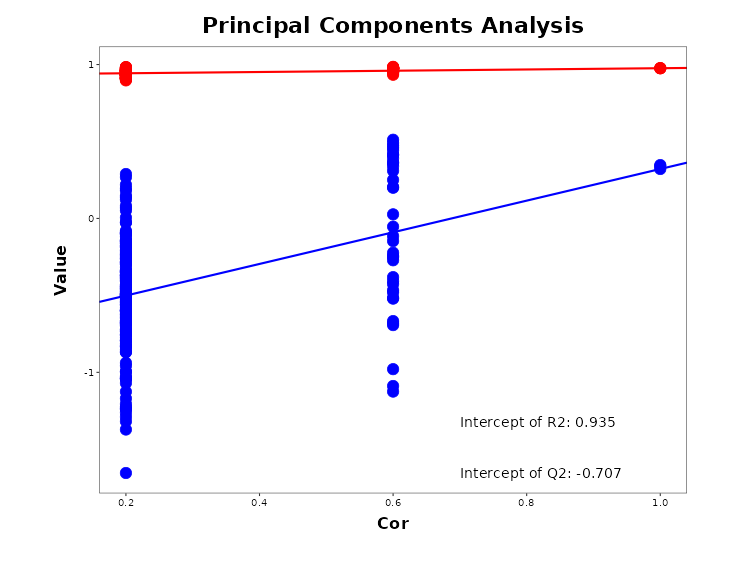


**Fig S6** Partial Least Squares Discriminant Analysis (PLS-DA) permutation testing plots between HDS and PDS group. HDS, healthy dairy sheep in postpartum; PDS, paretic dairy sheep in postpartum.


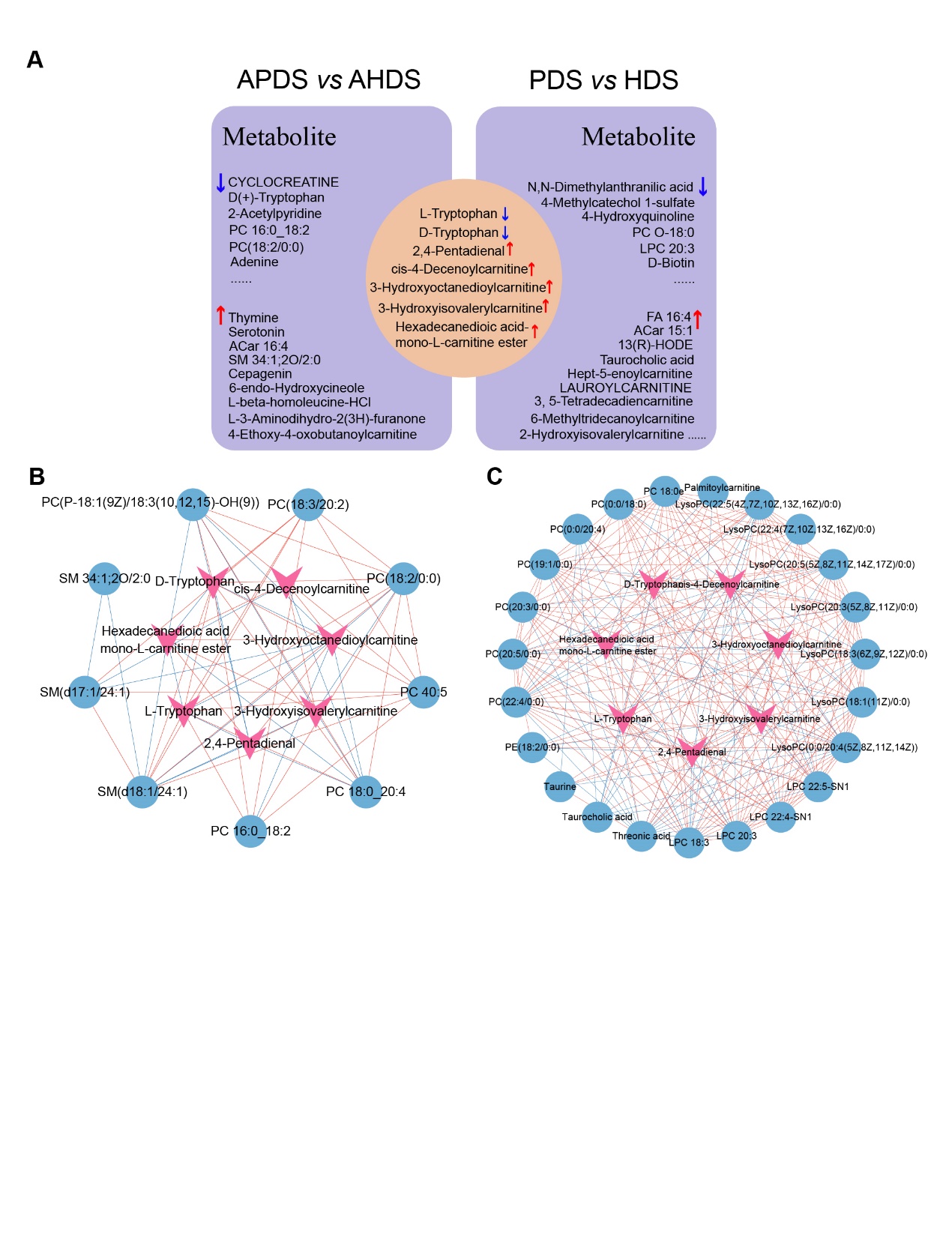


**Fig S7** Consistent metabolites were expressed in healthy and paretic sheep at different stages. **A** The differences and similarities of metabolite changes were in APDS vs AHDS and PDS vs HDS. **B** The correlation network diagram between shared metabolites and metabolites enriched in lipid metabolism in antepartum and postpartum.


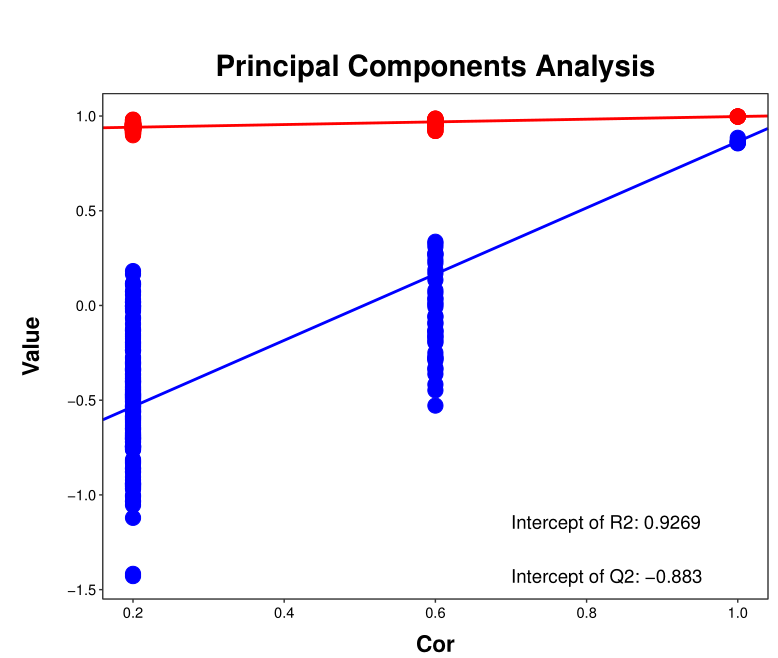


**Fig S8** Partial Least Squares Discriminant Analysis (PLS-DA) permutation testing plots between PDS and APDS group. PDS, paretic dairy sheep in postpartum; APDS, paretic dairy sheep in antepartum.


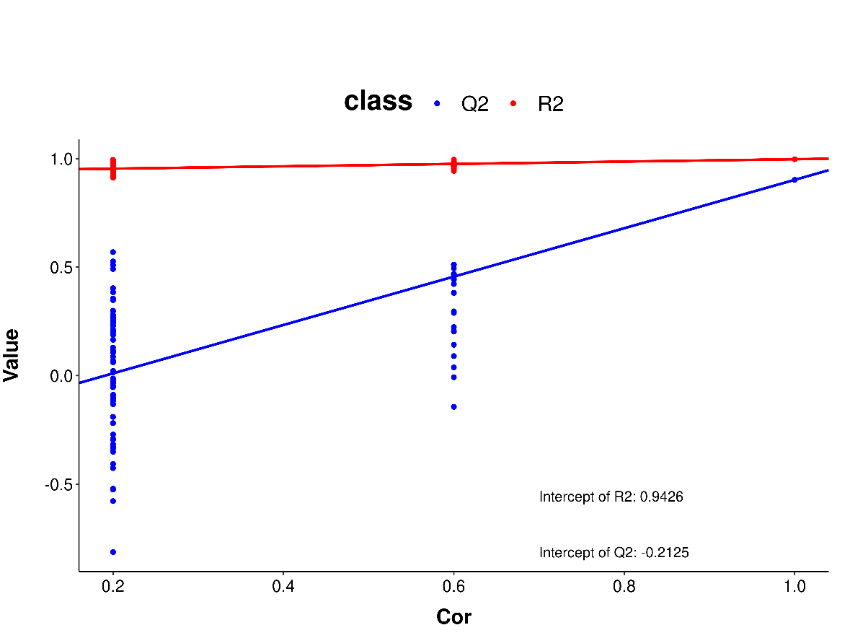


**Fig S9** Orthogonal Partial Least Squares Discriminant Analysis (OPLS-DA) permutation testing plots between PDS and APDS group. PDS, paretic dairy sheep in postpartum; APDS, paretic dairy sheep in antepartum.
