# Supplementary material for: Integrated multi-omics identifies dysregulated lipid metabolism of paresis in dairy sheep during the early transition period
Source: Microbiol Spectr. 2025 Oct 9;13(11):e01544-25. doi: 10.1128/spectrum.01544-25 (PMC12584643; doi:10.1128/spectrum.01544-25)
Supplement: Supplemental tables — Tables S1 to S10. [file spectrum.01544-25-s0002.docx]

**Additional table**

Table S1 Ingredient and chemical composition of experiment diets

| Items | Content |
| --- | --- |
| Ingredient composition, % of DM | |
| Alfalfa hay | 20.00 |
| Oat grass | 20.00 |
| Corn straw | 10.00 |
| Corn grain | 25.00 |
| Molasses | 3.00 |
| Corn germ meal | 6.00 |
| Soybean meal | 5.00 |
| Cottonseed meal | 8.00 |
| Limestone | 1.00 |
| NaCl | 0.50 |
| Extruded urea | 0.50 |
| Premix^1^ | 1.00 |
| Total | 100.00 |
| Nutrient composition^2^, % of DM | |
| DM, % | 87.60 |
| CP | 17.70 |
| EE | 2.61 |
| Ash | 35.45 |
| NDF | 22.29 |
| ADF | 1.00 |
| Ca | 0.40 |
| P | 20.32 |
| ME, MJ/kg | 10.40 |

^1^Premix provided (per kg) vitamin A, 100,000 IU; vitamin D, 30,000 IU; vitamin E, 500 IU; Fe, 1,500 mg; Zn, 2,000 mg; Mn, 1,000 mg; Cu, 800 mg; Se, 8 mg; Co, 8 mg; I, 15 mg.

^2^DM = dry matter, CP = crude protein, EE = ether extract, Ash = crude ash, NDF = neutral detergent fiber, ADF = acid detergent fiber, Ca = calcium, P = phosphorus, ME = metabolizable energy.

Table S2 Information of dairy sheep maternal and its offspring (n^1^ = 122)

| Items | Mean | Minimum | Maximum | SD^2^ |
| --- | --- | --- | --- | --- |
| Animal description | | | | |
| Age, months | 39.30 | 22.68 | 66.77 | 10.54 |
| Parity | 2.18 | 1.00 | 6.00 | 1.00 |
| Litter number | 2.33 | 1.00 | 5.00 | 0.94 |
| Survival number of offspring | 2.07 | 0.00 | 5.00 | 1.04 |
| Death number of offspring | 0.25 | 0.00 | 3.00 | 0.62 |
| Survival rate of offspring | 89.21 | 0.00 | 100.00 | 26.86 |
| Maternal weight during pregnancy, kg | | | | |
| 0 d | 87.63 | 52.65 | 124.74 | 12.00 |
| 30 d | 91.31 | 45.42 | 131.86 | 13.14 |
| 60 d | 96.61 | 53.35 | 125.24 | 13.16 |
| 90 d | 96.02 | 57.04 | 122.29 | 12.80 |
| Postpartum 1 d | 86.19 | 57.01 | 110.03 | 12.78 |
| Birth weight of offspring, kg | | | | |
| Litter weight | 7.65 | 2.50 | 14.60 | 3.32 |
| Average weight | 3.74 | 1.95 | 7.00 | 1.53 |

^1^n, number of observations in the data set.

^2^SD, standard deviation.

Table S3 Litter number and death number of offspring in healthy and paretic dairy sheep

| Items^1^ | HDS | PDS | SEM^2^ | P-value |
| --- | --- | --- | --- | --- |
| Litter number | 2.64 | 2.55 | 0.440 | 0.838 |
| Death number of offspring | 0.00 | 1.55 | 0.282 | <0.001 |

^1^HDS: healthy dairy sheep; PDS, paretic dairy sheep.

^2^SEM: Standard error of mean.

Table S4 Identification of different metabolites in plasma between APDS and AHDS group

| Metabolite | M/Z | Retention time | VIP | FC | P-value |
| --- | --- | --- | --- | --- | --- |
| Down-regulated | | | | | |
| D(+)-Tryptophan | 203.08 | 3.50 | 1.50 | 0.66 | 0.009 |
| L-Tryptophan | 188.07 | 3.50 | 1.48 | 0.64 | 0.015 |
| 3-Hydroxyhexadecadienoylcarnitine | 412.30 | 4.90 | 2.74 | 0.31 | 0.016 |
| 1_methyl_1H_indole_4_carboxylic_acid | 174.06 | 4.13 | 2.02 | 0.46 | 0.017 |
| 2-Acetylpyridine | 120.06 | 8.85 | 1.16 | 0.76 | 0.017 |
| D-Tryptophan | 205.10 | 3.50 | 1.43 | 0.66 | 0.018 |
| CYCLOCREATINE | 144.08 | 3.50 | 1.70 | 0.56 | 0.019 |
| Adenine | 136.06 | 9.93 | 1.20 | 0.74 | 0.020 |
| PC(18:2/0:0) | 520.34 | 9.98 | 1.03 | 0.80 | 0.021 |
| PC 16:0_18:2 | 816.58 | 7.66 | 1.23 | 0.73 | 0.023 |
| dodeca-2,8-dienedioic acid | 225.11 | 3.91 | 1.39 | 0.70 | 0.024 |
| 8-oxononanoic acid | 171.10 | 10.05 | 1.02 | 0.81 | 0.025 |
| Acetylenedicarboxylic acid | 112.99 | 1.12 | 1.91 | 0.55 | 0.026 |
| 4-amino-3-indol-3-ylazoline-2,5-dione | 227.08 | 3.50 | 1.47 | 0.62 | 0.028 |
| SM(d18:1/24:1) | 813.68 | 7.82 | 1.37 | 0.69 | 0.029 |
| Cyclo(Leu-Pro) | 211.14 | 3.69 | 1.41 | 0.68 | 0.030 |
| SM(d17:1/24:1) | 799.67 | 7.79 | 1.82 | 0.55 | 0.030 |
| PC 18:0_20:4 | 868.61 | 8.14 | 1.11 | 0.76 | 0.037 |
| L-Homoserine | 119.05 | 1.55 | 1.00 | 0.80 | 0.037 |
| PC(18:3/20:2) | 808.58 | 5.10 | 1.23 | 0.71 | 0.039 |
| PC 40:5 | 894.62 | 7.68 | 1.64 | 0.61 | 0.043 |
| 3-Azetidinecarboxylic acid | 102.06 | 0.20 | 1.49 | 0.65 | 0.048 |
| PC(P-18:1(9Z)/18:3(10,12,15)-OH(9)) | 782.57 | 5.20 | 1.49 | 0.63 | 0.049 |
| Up-regulated | | | | | |
| L-3-Aminodihydro-2(3H)-furanone | 102.06 | 2.05 | 1.16 | 1.28 | 0.005 |
| 2,4-Pentadienal | 81.03 | 3.56 | 2.58 | 3.26 | 0.007 |
| L-beta-homoleucine-HCl | 144.10 | 0.74 | 2.37 | 3.09 | 0.015 |
| Thymine | 127.05 | 3.36 | 1.43 | 1.52 | 0.017 |
| 3-Hydroxyisovalerylcarnitine | 262.16 | 1.49 | 1.60 | 1.71 | 0.024 |
| Cepagenin | 445.30 | 6.34 | 2.62 | 4.72 | 0.028 |
| Serotonin | 177.10 | 3.35 | 1.60 | 1.73 | 0.030 |
| 6-endo-Hydroxycineole | 171.15 | 4.68 | 1.14 | 1.33 | 0.030 |
| ACar 16:4 | 392.28 | 5.11 | 2.57 | 4.82 | 0.033 |
| SM 34:1;2O/2:0 | 731.60 | 6.44 | 1.91 | 2.15 | 0.035 |
| 4-Ethoxy-4-oxobutanoylcarnitine | 290.16 | 2.16 | 2.31 | 3.55 | 0.035 |
| Hexadecanedioic acid mono-L-carnitine ester | 430.32 | 4.52 | 1.84 | 2.18 | 0.039 |
| 3-Hydroxyoctanedioylcarnitine | 334.19 | 3.32 | 2.65 | 6.40 | 0.045 |
| cis-4-Decenoylcarnitine | 315.24 | 4.37 | 2.56 | 3.82 | 0.049 |

Abbreviations: FC, fold change, APDS group vs. AHDS group; VIP, variable importance in the projection; M/Z, mass-to-charge ratio.

Table S5 Pathway enrichment analysis of metabolites with significant differences in plasma between APDS and AHDS group

| Pathway_ID | KEGG_Level_1 | KEGG_Level_2 | Pathway_Name | Significant ID Number | Rich.  Factor | P.value |
| --- | --- | --- | --- | --- | --- | --- |
| ko05231 | Human Diseases | Cancer: overview | Choline metabolism in cancer | 6 | 0.546 | <0.001 |
| ko04723 | Organismal Systems | Nervous system | Retrograde endocannabinoid signaling | 6 | 0.316 | <0.001 |
| ko00591 | Metabolism | Lipid metabolism | Linoleic acid metabolism | 6 | 0.214 | <0.001 |
| ko00592 | Metabolism | Lipid metabolism | alpha-Linolenic acid metabolism | 6 | 0.136 | <0.001 |
| ko00564 | Metabolism | Lipid metabolism | Glycerophospholipid metabolism | 6 | 0.107 | <0.001 |
| ko00590 | Metabolism | Lipid metabolism | Arachidonic acid metabolism | 6 | 0.076 | <0.001 |
| ko04217 | Cellular Processes | Cell growth and death | Necroptosis | 3 | 0.300 | <0.001 |
| ko04071 | Environmental Information Processing | Signal transduction | Sphingolipid signaling pathway | 3 | 0.200 | <0.001 |
| ko00600 | Metabolism | Lipid metabolism | Sphingolipid metabolism | 3 | 0.086 | <0.001 |
| ko04361 | Organismal Systems | Development and regeneration | Axon regeneration | 2 | 0.286 | <0.001 |
| ko01100 | Metabolism | Global and overview maps | Metabolic pathways | 14 | 0.005 | 0.001 |
| ko07211 | Drug Development | Target-based classification: G protein-coupled receptors | Serotonin receptor agonists/antagonists | 1 | 1.000 | 0.002 |
| ko04726 | Organismal Systems | Nervous system | Serotonergic synapse | 2 | 0.048 | 0.005 |
| ko00260 | Metabolism | Amino acid metabolism | Glycine, serine and threonine metabolism | 2 | 0.042 | 0.006 |
| ko01110 | Metabolism | Global and overview maps | Biosynthesis of secondary metabolites | 11 | 0.005 | 0.007 |
| ko01232 | Metabolism | Global and overview maps | Nucleotide metabolism | 2 | 0.035 | 0.009 |
| ko00380 | Metabolism | Amino acid metabolism | Tryptophan metabolism | 2 | 0.024 | 0.018 |
| ko05143 | Human Diseases | Infectious disease: parasitic | African trypanosomiasis | 1 | 0.125 | 0.020 |
| ko04540 | Cellular Processes | Cellular community - eukaryotes | Gap junction | 1 | 0.091 | 0.027 |
| ko04721 | Organismal Systems | Nervous system | Synaptic vesicle cycle | 1 | 0.083 | 0.030 |
| ko01230 | Metabolism | Global and overview maps | Biosynthesis of amino acids | 2 | 0.016 | 0.039 |
| ko01063 | Metabolism | Chemical structure transformation maps | Biosynthesis of alkaloids derived from shikimate pathway | 2 | 0.014 | 0.048 |

Table S6 PICRUSt2 KEGG function prediction between PDS and HDS group

| Items | HDS | PDS | P-value |
| --- | --- | --- | --- |
| KEGG_level2 |  |  |  |
| Lipid Metabolism | 0.029 | 0.026 | 0.032 |
| Signaling Molecules and Interaction | 0.002 | 0.002 | 0.016 |
| Xenobiotics Biodegradation and Metabolism | 0.017 | 0.014 | 0.032 |
| KEGG_level3 |  |  |  |
| Alzheimer's disease | 0.001 | 0.001 | 0.008 |
| Arachidonic acid metabolism | <0.001 | <0.001 | 0.032 |
| Basal transcription factors | <0.001 | <0.001 | 0.016 |
| Benzoate degradation | 0.003 | 0.002 | 0.008 |
| Biosynthesis of unsaturated fatty acids | 0.002 | 0.001 | 0.008 |
| Butanoate metabolism | 0.007 | 0.006 | 0.032 |
| Caffeine metabolism | <0.001 | <0.001 | 0.032 |
| Carbohydrate metabolism | 0.001 | 0.002 | 0.032 |
| Citrate cycle (TCA cycle) | 0.008 | 0.007 | 0.016 |
| Endocytosis | <0.001 | <0.001 | 0.025 |
| Fatty acid metabolism | 0.004 | 0.003 | 0.008 |
| Fc gamma R-mediated phagocytosis | <0.001 | <0.001 | 0.025 |
| Fructose and mannose metabolism | 0.008 | 0.010 | 0.032 |
| Galactose metabolism | 0.006 | 0.008 | 0.016 |
| GnRH signaling pathway | <0.001 | <0.001 | 0.025 |
| Limonene and pinene degradation | 0.001 | <0.001 | 0.008 |
| Lysine degradation | 0.003 | 0.002 | 0.008 |
| Other transporters | 0.003 | 0.003 | 0.032 |
| Peroxisome | 0.002 | 0.002 | 0.016 |
| Polycyclic aromatic hydrocarbon degradation | <0.001 | <0.001 | 0.016 |
| Propanoate metabolism | 0.007 | 0.006 | 0.008 |
| Ribosome biogenesis in eukaryotes | 0.001 | <0.001 | 0.032 |
| Starch and sucrose metabolism | 0.008 | 0.010 | 0.008 |
| Styrene degradation | <0.001 | <0.001 | 0.032 |
| Synthesis and degradation of ketone bodies | 0.001 | <0.001 | 0.008 |
| Tryptophan metabolism | 0.003 | 0.002 | 0.008 |
| Tyrosine metabolism | 0.002 | 0.002 | 0.008 |
| Valine, leucine and isoleucine degradation | 0.004 | 0.003 | 0.008 |
| Vibrio cholerae infection | <0.001 | <0.001 | 0.016 |

Table S7 Identification of different metabolites in plasma between PDS and HDS group

| Metabolite | M/Z | Retention time | VIP | FC | P-value |
| --- | --- | --- | --- | --- | --- |
| Down-regulated | | | | | |
| N,N-Dimethylanthranilic acid | 166.08 | 0.93 | 2.35 | 0.18 | <0.001 |
| 4-Hydroxyquinoline | 146.06 | 3.48 | 1.53 | 0.44 | 0.003 |
| 4-Methylcatechol 1-sulfate | 203.00 | 3.79 | 2.71 | 0.10 | 0.003 |
| PC O-18:0 | 524.37 | 6.72 | 1.38 | 0.51 | 0.003 |
| LPC 20:3 | 604.36 | 6.33 | 2.44 | 0.12 | 0.004 |
| D-Biotin | 227.08 | 3.77 | 2.15 | 0.24 | 0.004 |
| 2-[(1S,2S,4aR,8aS)-1-hydroxy-4a  -methyl-8-methylidene-1,2,3,4,5,6,7,8a  -octahydronaphthalen-2-yl]prop-2-enoic acid | 249.15 | 4.74 | 1.84 | 0.29 | 0.005 |
| Lyso PC (22:6) | 568.34 | 5.63 | 2.14 | 0.18 | 0.005 |
| 3'-Methoxyacetophenone | 149.06 | 4.36 | 2.34 | 0.13 | 0.005 |
| Proline betaine | 144.10 | 0.96 | 1.37 | 0.51 | 0.005 |
| Quinol sulfate | 188.99 | 3.48 | 2.73 | 0.12 | 0.005 |
| 2-Methyl-1,2,3,4-tetrahydro-6,7-isoquinolinediol | 180.10 | 1.41 | 2.35 | 0.18 | 0.005 |
| PE(20:4/0:0) | 502.29 | 5.65 | 1.57 | 0.41 | 0.005 |
| Benzo[alpha]pyrene | 201.07 | 3.81 | 1.20 | 0.62 | 0.006 |
| LysoPC(20:3(5Z,8Z,11Z)/0:0) | 546.34 | 5.74 | 2.04 | 0.21 | 0.006 |
| 1-Oleoyl-sn-glycero-3-phosphocholine | 522.35 | 6.39 | 1.58 | 0.41 | 0.006 |
| Hippuric acid | 180.07 | 3.50 | 2.32 | 0.19 | 0.008 |
| m-Salicylic acid | 137.02 | 3.61 | 1.29 | 0.55 | 0.008 |
| 4-Acetyl-2-methylpyridine | 134.06 | 3.51 | 2.99 | 0.09 | 0.009 |
| LysoPC(18:3(6Z,9Z,12Z)/0:0) | 518.32 | 5.35 | 2.13 | 0.19 | 0.010 |
| arginine | 175.12 | 0.73 | 1.64 | 0.36 | 0.010 |
| salsolinol | 180.10 | 1.01 | 2.40 | 0.17 | 0.010 |
| LysoPC(18:1(11Z)/0:0) | 522.35 | 7.84 | 1.22 | 0.57 | 0.011 |
| 4-Allylphenol sulfate | 213.02 | 4.06 | 3.00 | 0.02 | 0.011 |
| LysoPC(22:5(4Z,7Z,10Z,13Z,16Z)/0:0) | 570.35 | 6.17 | 1.89 | 0.24 | 0.012 |
| PE(18:2/0:0) | 478.29 | 5.59 | 1.91 | 0.30 | 0.012 |
| LPC 18:3 | 576.33 | 5.37 | 2.37 | 0.11 | 0.012 |
| 1-(9Z-Octadecenoyl)-sn-glycero-3  -phosphocholine | 522.35 | 6.18 | 1.35 | 0.50 | 0.014 |
| 6-Quinolinol | 146.06 | 4.03 | 1.83 | 0.26 | 0.014 |
| PC(0:0/20:4) | 544.33 | 5.31 | 1.63 | 0.34 | 0.014 |
| 2-Acetylfuran | 109.03 | 3.48 | 1.71 | 0.31 | 0.015 |
| N,N'-Diphenylethylenediamine | 211.13 | 4.35 | 1.70 | 0.30 | 0.017 |
| 7-methyloctadecanoic acid | 297.28 | 6.45 | 1.75 | 0.28 | 0.017 |
| LysoPC(0:0/20:4(5Z,8Z,11Z,14Z)) | 544.34 | 5.70 | 2.04 | 0.24 | 0.018 |
| Scoulerin | 327.14 | 4.20 | 1.45 | 0.44 | 0.018 |
| PC(20:5/0:0) | 542.32 | 5.70 | 1.96 | 0.22 | 0.019 |
| 2-Ketobutyric acid | 101.02 | 1.50 | 1.01 | 0.67 | 0.020 |
| LysoPC(20:5(5Z,8Z,11Z,14Z,17Z)/0:0) | 542.32 | 5.34 | 1.72 | 0.29 | 0.020 |
| Pyrocatechol sulfate | 188.99 | 3.28 | 2.53 | 0.13 | 0.021 |
| Thymine | 127.05 | 3.36 | 1.75 | 0.25 | 0.021 |
| PC(0:0/18:0) | 524.37 | 10.00 | 1.19 | 0.55 | 0.021 |
| OXOPROLINE | 130.05 | 0.70 | 1.11 | 0.62 | 0.022 |
| LPC 22:5-SN1 | 570.35 | 5.92 | 1.78 | 0.25 | 0.023 |
| LysoPC(22:4(7Z,10Z,13Z,16Z)/0:0) | 572.36 | 5.92 | 1.86 | 0.21 | 0.024 |
| Xylitol | 175.12 | 1.51 | 1.61 | 0.33 | 0.025 |
| 5-beta-Androstane-3-alpha,17-beta-diol | 257.22 | 4.33 | 1.67 | 0.31 | 0.026 |
| PC(19:1/0:0) | 536.37 | 6.97 | 1.63 | 0.38 | 0.029 |
| PC(22:4/0:0) | 572.37 | 6.45 | 2.12 | 0.11 | 0.030 |
| Taurolithocholate | 482.29 | 4.51 | 2.17 | 0.21 | 0.030 |
| LPC 22:4-SN1 | 572.37 | 6.25 | 1.94 | 0.17 | 0.030 |
| L-(-)-3-Phenyllactic acid | 165.06 | 3.80 | 1.91 | 0.20 | 0.032 |
| PC 18:0e | 524.37 | 7.08 | 1.27 | 0.50 | 0.032 |
| Enterolactone | 299.13 | 3.82 | 1.86 | 0.29 | 0.034 |
| PC(20:3/0:0) | 546.35 | 6.10 | 1.83 | 0.20 | 0.035 |
| 2,8-Dihydroxyquinoline-beta-D-glucuronide | 338.09 | 3.43 | 2.43 | 0.06 | 0.038 |
| L-Tryptophan | 188.07 | 3.50 | 1.38 | 0.49 | 0.040 |
| 1-Decanoyl-2-hydroxy-sn-glycero-3  -phosphocholine | 412.24 | 4.40 | 2.27 | 0.18 | 0.041 |
| D-Tryptophan | 205.10 | 3.50 | 1.36 | 0.49 | 0.043 |
| 3-Indolepropionic acid | 188.07 | 4.32 | 1.83 | 0.20 | 0.043 |
| Up-regulated | | | | | |
| 13(R)-HODE | 295.23 | 7.42 | 1.83 | 2.99 | <0.001 |
| FA 16:4 | 247.17 | 6.57 | 2.09 | 4.01 | 0.001 |
| ACar 15:1 | 384.31 | 5.97 | 1.67 | 2.53 | 0.002 |
| 2,4-Pentadienal | 81.03 | 3.56 | 2.69 | 14.19 | 0.003 |
| 2-Hydroxyisovalerylcarnitine | 262.16 | 1.82 | 1.76 | 3.07 | 0.004 |
| Taurocholic acid | 1029.58 | 4.10 | 2.73 | 14.51 | 0.004 |
| LAUROYLCARNITINE | 344.28 | 5.08 | 2.48 | 5.88 | 0.006 |
| 3, 5-Tetradecadiencarnitine | 368.28 | 5.14 | 2.47 | 10.59 | 0.008 |
| Hept-5-enoylcarnitine | 272.19 | 3.90 | 1.51 | 2.26 | 0.008 |
| 6-Methyltridecanoylcarnitine | 372.31 | 5.99 | 2.22 | 4.44 | 0.008 |
| Non-3-enoylcarnitine | 300.22 | 4.19 | 1.73 | 2.87 | 0.009 |
| 2-Hexenoylcarnitine | 258.17 | 3.74 | 1.98 | 3.52 | 0.009 |
| 3-Methyltridecanoylcarnitine | 372.31 | 5.73 | 2.04 | 3.68 | 0.010 |
| Glyceraldehyde | 90.03 | 0.90 | 1.36 | 1.94 | 0.010 |
| O-(17-Carboxyheptadecanoyl)carnitine | 458.35 | 4.94 | 1.70 | 2.44 | 0.011 |
| 3-Methylglutarylcarnitine | 290.16 | 1.52 | 1.65 | 2.59 | 0.012 |
| Tridec-3-enoylcarnitine | 356.28 | 5.06 | 1.95 | 4.24 | 0.012 |
| 3-hydroxy-tetradecanoic acid | 243.20 | 5.32 | 1.12 | 1.60 | 0.013 |
| 6-Hydroxyheptanoylcarnitine | 290.20 | 3.69 | 1.71 | 2.77 | 0.016 |
| Octanoylcarnitine | 288.22 | 4.19 | 2.01 | 3.40 | 0.016 |
| Suberylglycine | 230.10 | 3.44 | 2.43 | 18.16 | 0.016 |
| C14Tetradecanoyl-L-Carnitine | 372.30 | 5.37 | 1.89 | 3.62 | 0.016 |
| 9-Hexadecenoylcarnitine | 398.33 | 6.36 | 2.16 | 3.94 | 0.016 |
| 7-Methylnonanoylcarnitine | 316.25 | 4.55 | 2.11 | 3.63 | 0.017 |
| Arabinofuranose | 151.06 | 10.08 | 1.39 | 2.18 | 0.017 |
| O-(13-Carboxytridecanoyl)carnitine | 402.28 | 4.30 | 1.96 | 3.45 | 0.018 |
| Butyrylcarnitine | 254.14 | 3.40 | 2.13 | 5.01 | 0.019 |
| Dl-3,4-Dihydroxymandelic acid | 149.02 | 1.29 | 1.20 | 1.72 | 0.019 |
| 6-(2-Hydroxyethoxy)-6-oxohexanoylcarnitine | 334.19 | 2.82 | 2.67 | 10.18 | 0.019 |
| Nonaethylene glycol | 432.28 | 3.74 | 1.39 | 2.23 | 0.020 |
| 3-HYDROXYANTHRANILATE | 154.06 | 0.86 | 1.06 | 1.59 | 0.021 |
| 3-Hydroxyhexanoylcarnitine | 276.18 | 3.50 | 2.07 | 3.39 | 0.023 |
| 3-Hydroxytetradecanoyl carnitine | 388.31 | 4.96 | 2.12 | 3.83 | 0.024 |
| 3-Hydroxyhexadecadienoylcarnitine | 412.30 | 4.90 | 2.16 | 4.22 | 0.024 |
| 4-Hydroxydecanoylcarnitine | 332.24 | 4.18 | 2.19 | 3.49 | 0.024 |
| 4-Methyloctanoylcarnitine | 302.23 | 4.30 | 2.14 | 3.54 | 0.025 |
| 4-Hydroxydodecanoylcarnitine | 360.27 | 4.50 | 2.23 | 3.71 | 0.027 |
| 4-Hydroxydecanedioylcarnitine | 362.22 | 3.59 | 2.13 | 5.20 | 0.027 |
| 7(14)-Bisabolene-2,3,10,11-tetrol | 271.19 | 5.02 | 1.75 | 4.19 | 0.028 |
| 4-Hydroxy-6-Methyl-2-Pyrone | 125.02 | 3.56 | 2.05 | 8.07 | 0.028 |
| O-ACETYLCARNITINE | 226.10 | 1.34 | 2.13 | 3.76 | 0.028 |
| Leucylhydroxyproline | 245.15 | 1.56 | 1.66 | 2.44 | 0.028 |
| beta-hydroxymyristic acid | 243.20 | 5.64 | 1.49 | 2.40 | 0.028 |
| 3-Methylpentanoylcarnitine | 260.19 | 3.85 | 1.87 | 2.75 | 0.028 |
| xi-gamma-Undecalactone | 183.14 | 5.57 | 1.62 | 2.48 | 0.029 |
| 5-Dodecenoylcarnitine | 342.26 | 4.80 | 1.81 | 2.89 | 0.029 |
| Acetyl-DL-carnitine | 204.12 | 3.18 | 1.82 | 3.60 | 0.030 |
| 4-hydroxybutanoic acid | 149.02 | 1.04 | 1.21 | 1.86 | 0.030 |
| 3-Octadec-9-enoyloxy-4  -(trimethylazaniumyl)butanoate | 426.36 | 10.01 | 1.56 | 2.42 | 0.031 |
| Palmitoylcarnitine | 400.34 | 10.01 | 1.70 | 2.74 | 0.032 |
| 2-methoxy-6-[(triphenylmethoxy)methyl]  -2H-3,4,5,6-tetrahydropyran-3,4,5-triol | 437.19 | 5.83 | 1.28 | 2.09 | 0.032 |
| 5-Hydroxyoctanoylcarnitine | 304.21 | 3.88 | 1.98 | 2.73 | 0.034 |
| 2-Carboxy-4-dodecanolide | 241.14 | 4.49 | 1.40 | 2.29 | 0.035 |
| Hexadecanedioic acid mono-L-carnitine ester | 430.32 | 4.52 | 1.74 | 2.81 | 0.035 |
| 3-hydroxynonanoyl carnitine | 318.23 | 4.00 | 1.68 | 2.37 | 0.036 |
| Acetyl-L-carnitine | 226.10 | 0.98 | 1.78 | 2.67 | 0.036 |
| tetradec-5-ynoic acid | 223.17 | 6.31 | 1.66 | 2.82 | 0.036 |
| 3-Hydroxyisovalerylcarnitine | 262.16 | 1.49 | 2.24 | 4.53 | 0.036 |
| 3-HYDROXY-3',4'-DIMETHOXYFLAVONE | 299.14 | 8.46 | 1.99 | 6.24 | 0.036 |
| 6-Methyldodecanoylcarnitine | 358.29 | 5.29 | 1.89 | 2.84 | 0.036 |
| Adipoyl-carnitine; AIF; CE0; CorrDec | 290.16 | 1.80 | 1.46 | 2.27 | 0.038 |
| 3-Hydroxyoctanedioylcarnitine | 334.19 | 3.32 | 2.15 | 7.43 | 0.038 |
| 3-Octenoylcarnitine | 286.20 | 4.06 | 1.81 | 2.70 | 0.038 |
| Valerylcarnitine | 246.17 | 3.61 | 1.36 | 2.04 | 0.039 |
| cis-4-Decenoylcarnitine | 315.24 | 4.37 | 1.87 | 2.76 | 0.039 |
| Taurine | 124.01 | 0.79 | 1.87 | 3.76 | 0.041 |
| N-(3-Acetamidopropyl)pyrrolidin-2-one | 185.13 | 3.52 | 1.54 | 3.28 | 0.042 |
| 2-AMINOPYRROLE | 83.06 | 9.55 | 1.04 | 1.47 | 0.042 |
| 11-Methyltetradecanoylcarnitine | 386.33 | 6.35 | 1.57 | 3.48 | 0.043 |
| Caroverine | 366.22 | 4.28 | 1.33 | 2.22 | 0.043 |
| Pro-Ile | 229.15 | 1.24 | 1.53 | 2.68 | 0.043 |
| 6-hydroxynon-3-enoic acid | 171.10 | 3.70 | 1.01 | 1.51 | 0.044 |
| Bilirubin | 585.27 | 8.69 | 1.82 | 4.92 | 0.045 |
| 10-Hydroxyheptadecanoylcarnitine | 430.35 | 6.07 | 2.12 | 4.06 | 0.045 |
| 5-Hydroxypentanoylcarnitine | 262.16 | 2.27 | 1.51 | 2.36 | 0.046 |
| Threonic acid | 135.03 | 0.78 | 1.44 | 2.94 | 0.046 |
| 3-methylheptanedioylcarnitine | 318.19 | 3.56 | 1.64 | 2.65 | 0.047 |
| Octadecanedioic acid | 313.24 | 6.00 | 1.80 | 3.31 | 0.049 |
| 5-Hydroxydec-7-enedioylcarnitine | 360.20 | 3.54 | 1.88 | 5.04 | 0.049 |

Abbreviations: FC, fold change, PDS group vs. HDS group; VIP, variable importance in the projection; M/Z, mass-to-charge ratio.

Table S8 Pathway enrichment analysis of metabolites with significant differences in plasma between PDS and HDS group

| Pathway_ID | KEGG_Level_1 | KEGG_Level_2 | Pathway_Name | Significant ID Number | Rich.  Factor | P.value |
| --- | --- | --- | --- | --- | --- | --- |
| ko05231 | Human Diseases | Cancer: overview | Choline metabolism in cancer | 18 | 1.636 | <0.001 |
| ko00564 | Metabolism | Lipid metabolism | Glycerophospholipid metabolism | 20 | 0.357 | <0.001 |
| ko04148 | Cellular Processes | Transport and catabolism | Efferocytosis | 11 | 0.611 | <0.001 |
| ko04723 | Organismal Systems | Nervous system | Retrograde endocannabinoid signaling | 9 | 0.474 | <0.001 |
| ko00591 | Metabolism | Lipid metabolism | Linoleic acid metabolism | 7 | 0.250 | <0.001 |
| ko00592 | Metabolism | Lipid metabolism | alpha-Linolenic acid metabolism | 7 | 0.159 | <0.001 |
| ko00590 | Metabolism | Lipid metabolism | Arachidonic acid metabolism | 7 | 0.089 | <0.001 |
| ko04136 | Cellular Processes | Transport and catabolism | Autophagy - other | 2 | 0.667 | <0.001 |
| ko04138 | Cellular Processes | Transport and catabolism | Autophagy - yeast | 2 | 0.667 | <0.001 |
| ko05130 | Human Diseases | Infectious disease: bacterial | Pathogenic Escherichia coli infection | 2 | 0.667 | <0.001 |
| ko00563 | Metabolism | Glycan biosynthesis and metabolism | Glycosylphosphatidylinositol (GPI)-anchor biosynthesis | 2 | 0.500 | <0.001 |
| ko05167 | Human Diseases | Infectious disease: viral | Kaposi sarcoma-associated herpesvirus infection | 2 | 0.400 | <0.001 |
| ko04140 | Cellular Processes | Transport and catabolism | Autophagy - animal | 2 | 0.333 | 0.001 |
| ko00430 | Metabolism | Metabolism of other amino acids | Taurine and hypotaurine metabolism | 2 | 0.083 | 0.009 |
| ko00400 | Metabolism | Amino acid metabolism | Phenylalanine, tyrosine and tryptophan biosynthesis | 2 | 0.057 | 0.019 |
| ko00120 | Metabolism | Lipid metabolism | Primary bile acid biosynthesis | 2 | 0.043 | 0.033 |
| ko00260 | Metabolism | Amino acid metabolism | Glycine, serine and threonine metabolism | 2 | 0.042 | 0.034 |
| ko04361 | Organismal Systems | Development and regeneration | Axon regeneration | 1 | 0.143 | 0.042 |
| ko05143 | Human Diseases | Infectious disease: parasitic | African trypanosomiasis | 1 | 0.125 | 0.048 |

Table S9 Identification of different metabolites in plasma between PDS and APDS group

| Metabolite | M/Z | Retention time | VIP | FC | P-value |
| --- | --- | --- | --- | --- | --- |
| Down-regulated | | | | | |
| 4-Pyridoxic acid | 182.05 | 3.23 | 1.38 | 0.43 | 0.009 |
| Homoarginine | 189.13 | 0.80 | 1.96 | 0.20 | 0.017 |
| L-Glutamic acid | 148.06 | 0.68 | 1.29 | 0.44 | 0.008 |
| Cholic acid | 407.28 | 4.80 | 2.23 | 0.11 | 0.010 |
| L-Tyrosine | 180.07 | 1.51 | 1.05 | 0.58 | 0.011 |
| m-Salicylic acid | 137.02 | 3.61 | 1.03 | 0.61 | 0.001 |
| Glycocholic acid | 464.30 | 4.15 | 2.12 | 0.05 | 0.026 |
| Taurolithocholate | 482.29 | 4.51 | 2.04 | 0.15 | 0.016 |
| 3-HYDROXYBUTANOIC ACID | 104.03 | 0.80 | 1.58 | 0.34 | 0.024 |
| 4-Hydroxyquinoline | 146.06 | 3.48 | 1.01 | 0.60 | 0.007 |
| 7Z, 10Z, 13Z, 16Z, 19Z-docosapentaenoic acid | 329.25 | 7.98 | 1.35 | 0.39 | 0.007 |
| 9a-hydroxy-3,4a,5-trimethyl-2H,4H,4aH,5H,6H,  7H,8H,8aH,9H,9aH-naphtho[2,3-b]furan-2-one | 249.15 | 5.26 | 1.31 | 0.42 | 0.022 |
| PE(18:1(9Z)/0:0) | 478.29 | 6.04 | 1.29 | 0.34 | 0.024 |
| 1-Hexadecanoyl-sn-glycero-3-phosphocholine | 496.34 | 6.15 | 1.68 | 0.30 | 0.000 |
| 11-methylnonadecanoic acid | 311.30 | 9.61 | 1.25 | 0.44 | 0.041 |
| 3,7R,11R,15-tetramethyl-hexadecanoic acid | 311.30 | 9.94 | 1.03 | 0.52 | 0.039 |
| 3'-Methoxyacetophenone | 149.06 | 4.36 | 1.26 | 0.39 | 0.017 |
| 1-heptadecanoyl-sn-glycero-3-phosphocholine | 510.35 | 6.71 | 2.01 | 0.15 | 0.000 |
| Arachidonic acid | 303.23 | 7.82 | 1.27 | 0.40 | 0.016 |
| Hippuric acid | 180.07 | 3.50 | 2.14 | 0.17 | 0.007 |
| Pregnanolone sulfate | 397.21 | 4.38 | 2.26 | 0.10 | 0.000 |
| 5alpha-Pregnan-3beta,20beta-diol 20-sulfate | 399.22 | 4.27 | 2.28 | 0.11 | 0.000 |
| LysoPC(P-16:0/0:0) | 480.34 | 6.47 | 1.46 | 0.35 | 0.003 |
| 2,4-Quinolinediol | 162.06 | 3.62 | 1.08 | 0.51 | 0.019 |
| Indolelactic acid | 204.07 | 3.80 | 1.42 | 0.39 | 0.047 |
| Lyso-PC(16:0) | 496.34 | 5.94 | 1.99 | 0.19 | 0.000 |
| cis-4,10,13,16-Docosatetraenoic Acid | 331.26 | 7.97 | 1.44 | 0.29 | 0.021 |
| 3-Indolepropionic acid | 188.07 | 4.32 | 1.90 | 0.17 | 0.003 |
| Lyso-PAF C-16 | 482.36 | 6.35 | 1.59 | 0.33 | 0.000 |
| LysoPE(16:0/0:0) | 454.29 | 5.80 | 1.68 | 0.26 | 0.001 |
| LPC O-18:1 | 508.38 | 6.96 | 1.72 | 0.27 | 0.000 |
| LPE 18:1 | 478.29 | 6.24 | 1.33 | 0.30 | 0.029 |
| PIPECOLATE | 130.09 | 0.97 | 1.01 | 0.49 | 0.043 |
| 2-[(1S,2S,4aR,8aS)-1-hydroxy-4a-methyl  -8-methylidene-1,2,3,4,5,6,7,8a-  octahydronaphthalen-2-yl]prop-2-enoic acid | 249.15 | 4.74 | 1.48 | 0.36 | 0.001 |
| 5-Methylcytosine | 112.05 | 1.50 | 1.47 | 0.39 | 0.003 |
| LPE 18:2 | 476.28 | 5.67 | 1.84 | 0.21 | 0.001 |
| Tyrosine | 180.07 | 1.14 | 1.35 | 0.43 | 0.022 |
| Uridine | 243.06 | 1.80 | 1.54 | 0.34 | 0.002 |
| 1-Stearoyl-2-hydroxy-sn-glycero-3-phosphocholine | 546.35 | 7.08 | 1.90 | 0.21 | 0.000 |
| Enterolactone | 299.13 | 3.82 | 1.49 | 0.34 | 0.042 |
| 1-Oleoyl-sn-glycero-3-phosphocholine | 522.35 | 6.39 | 1.69 | 0.27 | 0.001 |
| L-Glutamine | 145.06 | 0.79 | 1.06 | 0.58 | 0.008 |
| p-Acetaminobenzoic acid | 178.05 | 3.51 | 2.25 | 0.13 | 0.001 |
| N-Choloylglycine | 466.32 | 4.13 | 1.99 | 0.05 | 0.042 |
| Docosahexaenoic acid | 327.23 | 7.61 | 1.36 | 0.33 | 0.021 |
| LPC O-16:0 | 482.36 | 6.67 | 1.66 | 0.29 | 0.001 |
| Lyso PC (16:1) | 494.32 | 5.54 | 2.05 | 0.16 | 0.000 |
| LPC 16:0 | 540.33 | 5.96 | 2.17 | 0.14 | 0.000 |
| Serotonin | 177.10 | 3.35 | 1.83 | 0.25 | 0.016 |
| LysoPE(18:0/0:0) | 480.31 | 6.82 | 1.60 | 0.30 | 0.003 |
| LPC 18:1 | 566.35 | 6.42 | 2.00 | 0.18 | 0.000 |
| 4-Acetyl-2-methylpyridine | 134.06 | 3.51 | 2.70 | 0.08 | 0.009 |
| L-Tryptophan | 188.07 | 3.50 | 1.16 | 0.51 | 0.040 |
| 5(S)-HETE | 301.22 | 7.26 | 1.40 | 0.38 | 0.012 |
| 2-Amino-4-chloropyridine | 127.00 | 1.14 | 1.15 | 0.54 | 0.047 |
| Amino acids | 136.08 | 1.54 | 1.08 | 0.56 | 0.014 |
| trans-Cinnamic acid | 131.05 | 3.79 | 2.00 | 0.14 | 0.010 |
| D-Tryptophan | 205.10 | 3.50 | 1.14 | 0.52 | 0.043 |
| N,N'-Diphenylethylenediamine | 211.13 | 4.35 | 1.55 | 0.35 | 0.000 |
| 1-Palmitoyl-Sn-Glycero-3-Phosphocholine | 991.67 | 6.14 | 3.02 | 0.02 | 0.000 |
| Quinol sulfate | 188.99 | 3.48 | 2.30 | 0.13 | 0.007 |
| Pyrocatechol sulfate | 188.99 | 3.28 | 2.29 | 0.16 | 0.018 |
| LPC 18:0 | 582.38 | 7.11 | 2.01 | 0.18 | 0.001 |
| 4-Hydroxybenzaldehyde | 105.03 | 3.50 | 1.80 | 0.26 | 0.018 |
| LPC 18:2 | 578.35 | 5.71 | 2.17 | 0.13 | 0.000 |
| LPC 16:1 | 552.33 | 5.54 | 2.18 | 0.13 | 0.000 |
| 2-Acetylfuran | 109.03 | 3.48 | 1.54 | 0.36 | 0.000 |
| Lyso PC (22:6) | 568.34 | 5.63 | 2.11 | 0.13 | 0.000 |
| LPC 17:0 | 568.36 | 6.73 | 2.05 | 0.16 | 0.002 |
| 4-Methylcatechol 1-sulfate | 203.00 | 3.79 | 2.36 | 0.11 | 0.003 |
| 4-amino-3-indol-3-ylazoline-2,5-dione | 227.08 | 3.50 | 1.60 | 0.31 | 0.014 |
| PC(22:4/0:0) | 572.37 | 6.45 | 2.58 | 0.06 | 0.000 |
| salsolinol | 180.10 | 1.01 | 1.58 | 0.30 | 0.034 |
| PC(17:1/0:0) | 508.34 | 5.91 | 1.78 | 0.25 | 0.000 |
| Cryptolepine | 233.10 | 0.85 | 1.98 | 0.18 | 0.000 |
| 2,3-Diphenylpyrazine | 233.10 | 1.11 | 2.16 | 0.13 | 0.000 |
| PC(20:3/0:0) | 546.35 | 6.10 | 2.28 | 0.09 | 0.000 |
| 5,11,14-Eicosatrienoic acid | 305.24 | 7.82 | 1.29 | 0.39 | 0.019 |
| LPC 17:1 | 566.35 | 6.21 | 1.89 | 0.21 | 0.000 |
| LPC 14:0 | 526.32 | 5.33 | 2.17 | 0.13 | 0.000 |
| PC(0:0/20:4) | 544.33 | 5.31 | 1.74 | 0.27 | 0.000 |
| PE(16:0/0:0) | 452.28 | 6.00 | 1.74 | 0.26 | 0.000 |
| PC(17:0/0:0) | 510.35 | 6.49 | 1.52 | 0.35 | 0.003 |
| Cepagenin | 445.30 | 6.34 | 2.09 | 0.12 | 0.011 |
| 6-Quinolinol | 146.06 | 4.03 | 1.65 | 0.28 | 0.001 |
| LPC 20:3 | 604.36 | 6.33 | 2.76 | 0.04 | 0.000 |
| LPC 18:3 | 576.33 | 5.37 | 2.63 | 0.06 | 0.000 |
| PC(0:0/18:0) | 524.37 | 10.00 | 1.75 | 0.27 | 0.000 |
| PC(15:0/0:0) | 482.32 | 5.65 | 1.98 | 0.13 | 0.003 |
| LysoPC(0:0/18:0) | 546.35 | 7.47 | 1.83 | 0.23 | 0.001 |
| LysoPC(0:0/20:4(5Z,8Z,11Z,14Z)) | 544.34 | 5.70 | 2.29 | 0.10 | 0.002 |
| LysoPC(22:5(4Z,7Z,10Z,13Z,16Z)/0:0) | 570.35 | 6.17 | 2.18 | 0.13 | 0.000 |
| D-Biotin | 227.08 | 3.77 | 2.29 | 0.12 | 0.001 |
| 1-(9Z-Octadecenoyl)-sn-glycero-3-phosphocholine | 522.35 | 6.18 | 1.66 | 0.27 | 0.001 |
| LPE 20:4 | 500.28 | 5.59 | 1.52 | 0.25 | 0.012 |
| arginine | 175.12 | 0.73 | 1.42 | 0.32 | 0.012 |
| Asn Lys Arg Asp | 530.28 | 3.97 | 2.70 | 0.03 | 0.008 |
| LysoPC(18:1(11Z)/0:0) | 522.35 | 7.84 | 1.72 | 0.27 | 0.000 |
| PE(18:0/0:0) | 480.31 | 7.11 | 1.44 | 0.35 | 0.007 |
| LysoPC(20:3(5Z,8Z,11Z)/0:0) | 546.34 | 5.74 | 2.15 | 0.11 | 0.000 |
| LysoPC(20:5(5Z,8Z,11Z,14Z,17Z)/0:0) | 542.32 | 5.34 | 2.03 | 0.18 | 0.000 |
| LysoPC(18:3(6Z,9Z,12Z)/0:0) | 518.32 | 5.35 | 2.41 | 0.09 | 0.001 |
| 4-Allylphenol sulfate | 213.02 | 4.06 | 3.23 | 0.02 | 0.000 |
| PC(19:1/0:0) | 536.37 | 6.97 | 1.76 | 0.25 | 0.006 |
| LysoPC(22:4(7Z,10Z,13Z,16Z)/0:0) | 572.36 | 5.92 | 2.01 | 0.17 | 0.000 |
| 2-Methyl-5-(8,11-pentadecadienyl)-1,3-benzenediol | 329.24 | 7.61 | 1.47 | 0.32 | 0.010 |
| Hexadecanoyl-hydroxy-sn-glycerophosphocholine | 480.31 | 5.95 | 2.11 | 0.15 | 0.000 |
| Cytosine deoxyribonucleoside | 228.10 | 1.77 | 1.39 | 0.40 | 0.016 |
| 2-Methyl-1,2,3,4-tetrahydro-6,7-isoquinolinediol | 180.10 | 1.41 | 1.62 | 0.31 | 0.021 |
| LPC 22:4 | 630.38 | 6.46 | 2.78 | 0.04 | 0.000 |
| PE(20:4/0:0) | 502.29 | 5.65 | 1.26 | 0.37 | 0.024 |
| 2,8-Dihydroxyquinoline-beta-D-glucuronide | 338.09 | 3.43 | 2.61 | 0.06 | 0.001 |
| PC(20:5/0:0) | 542.32 | 5.70 | 1.88 | 0.20 | 0.002 |
| LPC 20:4 | 602.35 | 5.75 | 2.47 | 0.07 | 0.000 |
| LPC 17:0-SN1 | 510.35 | 6.23 | 1.28 | 0.44 | 0.003 |
| LPC 22:4-SN1 | 572.37 | 6.25 | 2.50 | 0.08 | 0.000 |
| Aflatoxin B1 dialcohol | 329.10 | 3.88 | 1.39 | 0.41 | 0.011 |
| LPC 17:1-SN1 | 508.34 | 6.24 | 1.33 | 0.28 | 0.049 |
| PI 36:4 | 857.52 | 7.79 | 1.12 | 0.46 | 0.024 |
| 1-Octadecanoyl-2-(5Z,8Z,11Z,14Z-eicosatetraenoyl)  -sn-glycero-3-phospho-(1'-myo-inositol) | 885.55 | 8.14 | 1.19 | 0.44 | 0.036 |
| PI 40:5 | 911.57 | 8.18 | 1.99 | 0.17 | 0.000 |
| Tetracosanedioic acid | 397.33 | 8.48 | 1.73 | 0.16 | 0.024 |
| Docosanedioic acid | 369.30 | 7.48 | 1.78 | 0.21 | 0.002 |
| 2'-Deoxycytidine | 228.10 | 2.12 | 1.58 | 0.36 | 0.046 |
| LPC 22:5-SN1 | 570.35 | 5.92 | 1.91 | 0.19 | 0.000 |
| Sulfolithocholylglycine | 512.27 | 3.92 | 2.27 | 0.02 | 0.028 |
| Asymmetric dimethylarginine | 203.15 | 1.35 | 1.27 | 0.48 | 0.011 |
| L-Homoserine | 119.05 | 1.55 | 1.40 | 0.41 | 0.037 |
| PI 38:5 | 883.53 | 7.85 | 1.21 | 0.47 | 0.014 |
| Cryptotanshinone | 319.14 | 3.39 | 1.59 | 0.33 | 0.030 |
| Trp Gln | 333.15 | 3.51 | 2.05 | 0.15 | 0.004 |
| PC 18:0e | 524.37 | 7.08 | 1.88 | 0.22 | 0.000 |
| LPC 22:5 | 628.36 | 6.19 | 2.45 | 0.08 | 0.000 |
| Lumichrome | 241.08 | 3.37 | 1.16 | 0.52 | 0.002 |
| 2-amino-4-hydroxypyrimidine-5-carboxylic acid | 156.04 | 1.15 | 1.26 | 0.49 | 0.004 |
| Indole-5,6-quinone | 148.04 | 1.42 | 1.15 | 0.52 | 0.018 |
| Phosphatidylcholine lyso 16:0 | 554.35 | 5.96 | 2.10 | 0.16 | 0.000 |
| Phosphatidylcholine lyso 18:1 | 580.36 | 6.20 | 1.81 | 0.21 | 0.001 |
| Phosphatidylcholine lyso 17:0 | 568.36 | 6.50 | 1.65 | 0.30 | 0.012 |
| Xylitol | 175.12 | 1.51 | 1.56 | 0.28 | 0.005 |
| PC O-18:0 | 524.37 | 6.72 | 1.77 | 0.26 | 0.000 |
| Up-regulated |  |  |  |  |  |
| Threonic acid | 135.03 | 0.78 | 1.34 | 3.32 | 0.031 |
| LAUROYLCARNITINE | 344.28 | 5.08 | 2.25 | 8.66 | 0.004 |
| Tridec-3-enoylcarnitine | 356.28 | 5.06 | 2.32 | 11.42 | 0.024 |
| C14Tetradecanoyl-L-Carnitine | 372.30 | 5.37 | 2.27 | 10.81 | 0.000 |
| 6-Methyldodecanoylcarnitine | 358.29 | 5.29 | 2.21 | 8.15 | 0.007 |
| 4,8 Dimethylnonanoyl carnitine | 330.26 | 4.68 | 1.46 | 3.26 | 0.010 |
| Taurine | 124.01 | 0.79 | 1.95 | 6.47 | 0.002 |
| 5-Dodecenoylcarnitine | 342.26 | 4.80 | 2.29 | 9.35 | 0.002 |
| 7-Methylnonanoylcarnitine | 316.25 | 4.55 | 2.51 | 11.16 | 0.019 |
| 3-Octenoylcarnitine | 286.20 | 4.06 | 2.09 | 6.75 | 0.004 |
| 4-Hydroxy-6-Methyl-2-Pyrone | 125.02 | 3.56 | 2.00 | 11.52 | 0.008 |
| 7-Hydroxyoctanoylcarnitine | 304.21 | 3.60 | 2.22 | 8.75 | 0.000 |
| 4-Methyloctanoylcarnitine | 302.23 | 4.30 | 2.80 | 11.80 | 0.019 |
| 2-Hexenoylcarnitine | 258.17 | 3.74 | 1.89 | 5.12 | 0.000 |
| Non-3-enoylcarnitine | 300.22 | 4.19 | 1.93 | 5.33 | 0.002 |
| 3-Methylpentanoylcarnitine | 260.19 | 3.85 | 1.84 | 4.30 | 0.004 |
| Palmitoylcarnitine | 400.34 | 10.01 | 1.69 | 4.12 | 0.008 |
| xi-gamma-Undecalactone | 183.14 | 5.57 | 1.63 | 3.44 | 0.001 |
| N,N,N-TRIMETHYLLYSINE | 189.16 | 0.73 | 2.98 | 46.57 | 0.000 |
| 2,4-Pentadienal | 81.03 | 3.56 | 2.25 | 16.07 | 0.003 |
| p-Menthan-trans-2,5-diol | 171.14 | 5.52 | 1.55 | 2.95 | 0.032 |
| Sebacate | 201.11 | 4.16 | 1.52 | 3.54 | 0.007 |
| 2-methoxy-6-[(triphenylmethoxy)methyl]  -2H-3,4,5,6-tetrahydropyran-3,4,5-triol | 437.19 | 5.83 | 1.35 | 2.73 | 0.008 |
| xml:space=preserve>3-Methyl-L-Histidine&#12288; | 214.06 | 0.86 | 1.12 | 1.93 | 0.007 |
| 3-oxotetradecanoic acid | 241.18 | 5.78 | 1.18 | 2.43 | 0.039 |
| N,N-dimethyl-proline-proline betaine | 241.15 | 1.84 | 1.70 | 3.48 | 0.000 |
| N-Acetylcytidine | 286.10 | 3.37 | 1.03 | 1.76 | 0.047 |
| Cnidiol C | 169.12 | 5.18 | 1.52 | 3.37 | 0.006 |
| Octanoylcarnitine | 288.22 | 4.19 | 1.90 | 4.61 | 0.036 |
| 11-Methyltetradecanoylcarnitine | 386.33 | 6.35 | 2.08 | 9.45 | 0.002 |
| Valerylcarnitine | 246.17 | 3.61 | 1.50 | 2.96 | 0.001 |
| 1,11-Undecanedicarboxylic acid | 243.16 | 4.64 | 1.30 | 2.72 | 0.016 |
| Isobutyryl carnitine | 232.15 | 3.40 | 1.93 | 6.22 | 0.002 |
| 2-phenylfuro[2,3-h]chromen-4-one | 263.07 | 3.35 | 2.88 | 144.12 | 0.014 |
| 3-Hydroxydodecanoic acid | 215.17 | 4.53 | 1.47 | 3.38 | 0.016 |
| 5-Hydroxyoctanoylcarnitine | 304.21 | 3.88 | 1.97 | 5.53 | 0.003 |
| 9-Hexadecenoylcarnitine | 398.33 | 6.36 | 2.01 | 5.70 | 0.013 |
| Propionylcarnitine | 218.14 | 1.55 | 1.44 | 2.73 | 0.002 |
| 3-Hydroxytetradecanoyl carnitine | 388.31 | 4.96 | 2.42 | 13.35 | 0.001 |
| 4-Hydroxydodecanoylcarnitine | 360.27 | 4.50 | 2.36 | 11.32 | 0.005 |
| 6-Methyltridecanoylcarnitine | 372.31 | 5.99 | 2.09 | 7.00 | 0.007 |
| Acetylcarnitine | 204.12 | 1.33 | 1.88 | 4.94 | 0.001 |
| 4-Hydroxydecanoylcarnitine | 332.24 | 4.18 | 1.98 | 5.68 | 0.005 |
| 8-Hydroxydodecanoylcarnitine | 360.27 | 4.15 | 2.13 | 6.99 | 0.021 |
| 3-Methyltridecanoylcarnitine | 372.31 | 5.73 | 2.14 | 7.44 | 0.005 |
| 3-hydroxynonanoyl carnitine | 318.23 | 4.00 | 1.81 | 4.53 | 0.007 |
| trimethylammoinimbutyrate (TMAB) | 146.12 | 0.79 | 1.75 | 4.18 | 0.002 |
| 3, 5-Tetradecadiencarnitine | 368.28 | 5.14 | 2.16 | 9.23 | 0.009 |
| Hept-5-enoylcarnitine | 272.19 | 3.90 | 1.88 | 4.77 | 0.000 |
| Nonaethylene glycol | 432.28 | 3.74 | 1.11 | 2.14 | 0.026 |
| 10-Hydroxyheptadecanoylcarnitine | 430.35 | 6.07 | 3.17 | 66.29 | 0.000 |
| 3-Hydroxyisovalerylcarnitine | 262.16 | 1.49 | 1.37 | 2.41 | 0.001 |
| Undecanedioic acid | 215.13 | 4.32 | 1.33 | 2.84 | 0.017 |
| 6-Hydroxyheptanoylcarnitine | 290.20 | 3.69 | 1.94 | 5.54 | 0.000 |
| 2-Hydroxydecanoylcarnitine | 332.24 | 3.86 | 2.27 | 10.34 | 0.000 |
| 3-HYDROXYANTHRANILATE | 154.06 | 0.86 | 1.02 | 1.78 | 0.016 |
| 3-Octadec-9-enoyloxy-4-(trimethylazaniumyl)butanoate | 426.36 | 10.01 | 1.55 | 3.26 | 0.008 |
| Dodec-3-enedioic acid | 227.13 | 4.35 | 1.23 | 3.06 | 0.047 |
| ACar 15:1 | 384.31 | 5.97 | 1.60 | 3.21 | 0.001 |
| Leucylhydroxyproline | 245.15 | 1.56 | 1.94 | 5.51 | 0.000 |
| 3-hydroxypentadecanoyl carnitine | 402.32 | 5.19 | 2.96 | 36.48 | 0.001 |
| Adipoyl-carnitine; AIF; CE0; CorrDec | 290.16 | 1.80 | 1.74 | 3.95 | 0.014 |
| 3-Oxoheptanoylcarnitine | 288.18 | 3.58 | 2.23 | 9.36 | 0.000 |
| dodeca-2,9-dienedioic acid | 225.11 | 4.30 | 1.62 | 5.63 | 0.019 |
| Acetyl-L-carnitine | 226.10 | 0.98 | 2.02 | 6.21 | 0.000 |
| Dimethylguanidino valeric acid | 202.12 | 1.62 | 1.77 | 4.95 | 0.003 |
| L-Acetylcarnitine | 205.13 | 0.99 | 2.10 | 7.25 | 0.000 |
| Hexadecanedioic acid mono-L-carnitine ester | 430.32 | 4.52 | 1.91 | 6.05 | 0.001 |
| 3-hydroxytridecanoyl carnitine | 374.29 | 4.65 | 1.62 | 3.23 | 0.015 |
| CAR 7:0 | 274.20 | 3.95 | 2.18 | 7.83 | 0.000 |
| 5-Hydroxypentanoylcarnitine | 262.16 | 2.27 | 1.55 | 3.37 | 0.003 |
| O-(17-Carboxyheptadecanoyl)carnitine | 458.35 | 4.94 | 1.73 | 3.61 | 0.006 |
| 2-Hydroxycampholonic acid | 199.10 | 4.08 | 1.77 | 8.35 | 0.020 |
| Pro Ile | 229.15 | 2.24 | 2.00 | 7.70 | 0.003 |
| O-(11-Carboxyundecanoyl)carnitine | 374.25 | 4.02 | 1.88 | 6.00 | 0.002 |
| 3-Hydroxyhexadecadienoylcarnitine | 412.30 | 4.90 | 2.31 | 9.81 | 0.001 |
| 2-Hydroxyisovalerylcarnitine | 262.16 | 1.82 | 1.26 | 2.44 | 0.013 |
| FA 16:4 | 247.17 | 6.57 | 1.81 | 4.42 | 0.000 |
| 7-[(1R,2R,3R,5S)-3,5-Dihydroxy-2-  [(3S)-3-hydroxyoctyl]cyclopentyl]heptanoylcarnitine | 502.37 | 4.84 | 2.93 | 30.32 | 0.001 |
| Undecanedioylcarnitine | 360.24 | 3.91 | 1.98 | 7.39 | 0.002 |
| 4-aminovaleric acid betaine | 160.13 | 0.98 | 1.45 | 2.91 | 0.003 |
| Dodec-7-enedioylcarnitine | 372.24 | 3.96 | 2.57 | 20.57 | 0.001 |
| 13(R)-HODE | 295.23 | 7.42 | 1.67 | 3.54 | 0.001 |
| 3-hydroxyundecanoyl carnitine | 346.26 | 4.30 | 1.19 | 2.56 | 0.042 |
| cis-4-Decenoylcarnitine | 315.24 | 4.37 | 2.28 | 8.64 | 0.002 |
| Sebacoyl-L-carnitine | 346.22 | 3.80 | 2.38 | 14.51 | 0.000 |
| 4-Oxodecanoylcarnitine | 330.23 | 3.80 | 2.26 | 9.12 | 0.000 |
| 6-Hydroxydodecanedioylcarnitine | 390.25 | 3.89 | 1.08 | 2.15 | 0.039 |
| 4-Hydroxydecanedioylcarnitine | 362.22 | 3.59 | 2.56 | 26.41 | 0.002 |
| Tridec-8-enedioylcarnitine | 386.25 | 4.06 | 2.16 | 10.00 | 0.001 |
| 3-Hydroxyhexanoylcarnitine | 276.18 | 3.50 | 2.18 | 7.59 | 0.001 |
| Pro-Leu | 229.15 | 1.83 | 2.05 | 6.92 | 0.006 |
| Undec-5-enedioylcarnitine | 358.22 | 3.85 | 2.59 | 27.45 | 0.001 |
| 3-Hydroxydodeca-6,9-dienoylcarnitine | 356.24 | 4.27 | 2.18 | 9.31 | 0.000 |
| 1-METHYL-L-HISTIDINE | 192.07 | 0.89 | 1.33 | 2.22 | 0.001 |
| PC 40:5 | 894.62 | 7.68 | 1.08 | 1.97 | 0.029 |
| 2-Chloro-1-propanol | 93.01 | 6.54 | 1.04 | 1.76 | 0.006 |
| ACar 16:4 | 392.28 | 5.11 | 2.95 | 35.02 | 0.000 |
| 1-(2,4,5-Trimethoxyphenyl)-1,2-propanedione | 239.09 | 3.69 | 1.39 | 3.80 | 0.036 |
| 2,2-dimethylpentanedioylcarnitine | 304.18 | 3.42 | 1.66 | 3.74 | 0.008 |
| Nonanedioylcarnitine | 332.21 | 3.68 | 2.43 | 14.62 | 0.000 |
| Budralazine | 241.15 | 1.21 | 1.64 | 3.36 | 0.000 |
| 4-Methylvaleric acid | 115.00 | 1.23 | 1.06 | 1.97 | 0.036 |
| Non-6-enedioylcarnitine | 330.19 | 3.63 | 2.59 | 23.53 | 0.001 |
| 3-oxodecanoylcarnitine | 330.23 | 4.04 | 2.29 | 11.80 | 0.001 |
| METHACHOLINE | 160.13 | 1.25 | 1.22 | 2.23 | 0.008 |
| 2-Hydroxyundec-8-enoylcarnitine | 344.24 | 3.91 | 1.43 | 2.83 | 0.003 |
| 3-methylheptanedioylcarnitine | 318.19 | 3.56 | 2.19 | 8.27 | 0.002 |
| 3-Hydroxyoctanedioylcarnitine | 334.19 | 3.32 | 2.87 | 55.67 | 0.001 |
| 3-Hydroxyheptanoylcarnitine | 290.20 | 3.47 | 2.23 | 9.56 | 0.000 |
| O-(13-Carboxytridecanoyl)carnitine | 402.28 | 4.30 | 2.18 | 8.34 | 0.000 |
| Acetyl-DL-carnitine | 204.12 | 3.18 | 1.81 | 5.81 | 0.006 |
| 5-Hydroxydec-7-enedioylcarnitine | 360.20 | 3.54 | 2.72 | 33.28 | 0.001 |
| 5-Hydroxytrtradeca-7,9-dienoylcarnitine | 384.27 | 4.58 | 2.53 | 14.96 | 0.000 |
| 5-Hydroxyindole | 134.06 | 4.35 | 1.19 | 2.15 | 0.015 |
| Gluconate | 219.05 | 0.68 | 1.67 | 4.44 | 0.007 |
| O-ACETYLCARNITINE | 226.10 | 1.34 | 2.09 | 5.69 | 0.026 |
| Butyrylcarnitine | 254.14 | 3.40 | 2.33 | 13.94 | 0.001 |
| 5-Hydroxyindole-3-acetic acid | 192.07 | 3.66 | 1.50 | 2.93 | 0.012 |
| Tryptophanamide | 202.11 | 3.62 | 1.70 | 8.64 | 0.037 |
| N-(3-Acetamidopropyl)pyrrolidin-2-one | 185.13 | 3.52 | 1.31 | 3.27 | 0.040 |
| 6-(2-Hydroxyethoxy)-6-oxohexanoylcarnitine | 334.19 | 2.82 | 2.82 | 15.64 | 0.016 |
| L-Asparagine | 117.03 | 3.91 | 1.74 | 4.18 | 0.006 |
| Kamahine C | 267.12 | 4.38 | 1.35 | 2.81 | 0.015 |
| 10-Hydroxydecanoic Acid | 187.13 | 4.45 | 1.62 | 2.88 | 0.047 |
| Dec-5-enedioylcarnitine | 344.21 | 4.03 | 1.53 | 3.52 | 0.008 |
| Betaine-Aldehyde | 102.09 | 3.85 | 1.04 | 2.08 | 0.047 |
| 2-Deoxycytidine | 250.07 | 2.11 | 2.14 | 49.37 | 0.029 |
| 4-hydroxybutanoic acid | 149.02 | 1.04 | 1.08 | 1.95 | 0.028 |
| 3-Hydroxy-cis-5-octenoylcarnitine | 302.20 | 3.74 | 3.07 | 70.43 | 0.000 |
| Bicifadine | 174.12 | 1.57 | 1.96 | 12.72 | 0.026 |
| Caryophyllen-beta | 290.16 | 2.70 | 2.01 | 6.23 | 0.005 |
| 8-Oxodecanoylcarnitine | 330.23 | 4.27 | 2.14 | 7.36 | 0.002 |
| 3-Methylglutarylcarnitine | 290.16 | 1.52 | 1.80 | 4.20 | 0.021 |
| 4-Ethoxy-4-oxobutanoylcarnitine | 290.16 | 2.16 | 1.85 | 5.07 | 0.006 |
| 3-Hydroxydodec-6-enedioylcarnitine | 388.23 | 3.79 | 2.82 | 39.55 | 0.003 |
| Pro-Ile | 229.15 | 1.24 | 1.87 | 6.02 | 0.003 |
| Propionyl-L-carnitine | 218.14 | 1.23 | 1.25 | 2.18 | 0.003 |
| Arabinofuranose | 151.06 | 10.08 | 1.07 | 1.98 | 0.035 |
| 3-Guanidinopropanoate | 132.08 | 0.79 | 1.10 | 1.82 | 0.003 |
| Epiyangambin | 445.19 | 4.08 | 3.02 | 311.53 | 0.009 |

Abbreviations: FC, fold change, PDS group vs. APDS group; VIP, variable importance in the projection; M/Z, mass-to-charge ratio.

Table S10 Pathway enrichment analysis of metabolites with significant differences in plasma between PDS and APDS group

| Pathway_ID | KEGG_Level_1 | KEGG_Level_2 | Pathway_Name | Significant ID Number | Rich.  Factor | P.value |
| --- | --- | --- | --- | --- | --- | --- |
| ko04148 | Cellular Processes | Transport and catabolism | Efferocytosis | 29 | 1.611 | <0.001 |
| ko05231 | Human Diseases | Cancer: overview | Choline metabolism in cancer | 40 | 3.636 | <0.001 |
| ko00563 | Metabolism | Glycan biosynthesis and metabolism | Glycosylphosphatidylinositol (GPI)-anchor biosynthesis | 6 | 1.500 | <0.001 |
| ko04136 | Cellular Processes | Transport and catabolism | Autophagy - other | 6 | 2.000 | <0.001 |
| ko04138 | Cellular Processes | Transport and catabolism | Autophagy - yeast | 6 | 2.000 | <0.001 |
| ko00571 | Metabolism | Glycan biosynthesis and metabolism | Lipoarabinomannan (LAM) biosynthesis | 3 | 1.500 | <0.001 |
| ko00564 | Metabolism | Lipid metabolism | Glycerophospholipid metabolism | 52 | 0.929 | <0.001 |
| ko04723 | Organismal Systems | Nervous system | Retrograde endocannabinoid signaling | 17 | 0.895 | <0.001 |
| ko00591 | Metabolism | Lipid metabolism | Linoleic acid metabolism | 12 | 0.429 | <0.001 |
| ko04140 | Cellular Processes | Transport and catabolism | Autophagy - animal | 6 | 1.000 | <0.001 |
| ko00592 | Metabolism | Lipid metabolism | alpha-Linolenic acid metabolism | 11 | 0.250 | <0.001 |
| ko00590 | Metabolism | Lipid metabolism | Arachidonic acid metabolism | 13 | 0.165 | <0.001 |
| ko05230 | Human Diseases | Cancer: overview | Central carbon metabolism in cancer | 7 | 0.189 | <0.001 |
| ko05030 | Human Diseases | Substance dependence | Cocaine addiction | 4 | 0.571 | <0.001 |
| ko05130 | Human Diseases | Infectious disease: bacterial | Pathogenic Escherichia coli infection | 3 | 1.000 | <0.001 |
| ko04974 | Organismal Systems | Digestive system | Protein digestion and absorption | 7 | 0.149 | <0.001 |
| ko05031 | Human Diseases | Substance dependence | Amphetamine addiction | 4 | 0.444 | <0.001 |
| ko05034 | Human Diseases | Substance dependence | Alcoholism | 4 | 0.400 | <0.001 |
| ko00970 | Genetic-Information Processing | Translation | Aminoacyl-tRNA biosynthesis | 7 | 0.135 | <0.001 |
| ko05167 | Human Diseases | Infectious disease: viral | Kaposi sarcoma-associated herpesvirus infection | 3 | 0.600 | <0.001 |
| ko05132 | Human Diseases | Infectious disease: bacterial | Salmonella infection | 3 | 0.600 | <0.001 |
| ko04724 | Organismal Systems | Nervous system | Glutamatergic synapse | 3 | 0.375 | <0.001 |
| ko02010 | Environmental Information Processing | Membrane transport | ABC transporters | 9 | 0.065 | <0.001 |
| ko01060 | Metabolism | Chemical structure transformation maps | Biosynthesis of plant secondary metabolites | 9 | 0.064 | <0.001 |
| ko04727 | Organismal Systems | Nervous system | GABAergic synapse | 3 | 0.333 | <0.001 |
| ko04730 | Organismal Systems | Nervous system | Long-term depression | 3 | 0.333 | <0.001 |
| ko05152 | Human Diseases | Infectious disease: bacterial | Tuberculosis | 3 | 0.333 | <0.001 |
| ko04540 | Cellular Processes | Cellular community - eukaryotes | Gap junction | 3 | 0.273 | <0.001 |
| ko01230 | Metabolism | Global and overview maps | Biosynthesis of amino acids | 8 | 0.063 | 0.001 |
| ko04721 | Organismal Systems | Nervous system | Synaptic vesicle cycle | 3 | 0.250 | 0.001 |
| ko00250 | Metabolism | Amino acid metabolism | Alanine, aspartate and glutamate metabolism | 4 | 0.143 | 0.001 |
| ko00400 | Metabolism | Amino acid metabolism | Phenylalanine, tyrosine and tryptophan biosynthesis | 4 | 0.114 | 0.002 |
| ko04964 | Organismal Systems | Excretory system | Proximal tubule bicarbonate reclamation | 3 | 0.177 | 0.002 |
| ko04068 | Environmental Information Processing | Signal transduction | FoxO signaling pathway | 2 | 0.400 | 0.002 |
| ko00240 | Metabolism | Nucleotide metabolism | Pyrimidine metabolism | 5 | 0.078 | 0.003 |
| ko00910 | Metabolism | Energy metabolism | Nitrogen metabolism | 3 | 0.158 | 0.003 |
| ko00997 | Metabolism | Biosynthesis of other secondary metabolites | Biosynthesis of various other secondary metabolites | 5 | 0.075 | 0.003 |
| ko05016 | Human Diseases | Neurodegenerative disease | Huntington disease | 2 | 0.333 | 0.003 |
| ko04916 | Organismal Systems | Endocrine system | Melanogenesis | 2 | 0.333 | 0.003 |
| ko04726 | Organismal Systems | Nervous system | Serotonergic synapse | 4 | 0.095 | 0.003 |
| ko04720 | Organismal Systems | Nervous system | Long-term potentiation | 2 | 0.286 | 0.004 |
| ko05017 | Human Diseases | Neurodegenerative disease | Spinocerebellar ataxia | 2 | 0.286 | 0.004 |
| ko05033 | Human Diseases | Substance dependence | Nicotine addiction | 2 | 0.286 | 0.004 |
| ko04361 | Organismal Systems | Development and regeneration | Axon regeneration | 2 | 0.286 | 0.004 |
| ko00220 | Metabolism | Amino acid metabolism | Arginine biosynthesis | 3 | 0.130 | 0.005 |
| ko00340 | Metabolism | Amino acid metabolism | Histidine metabolism | 4 | 0.085 | 0.005 |
| ko00650 | Metabolism | Carbohydrate metabolism | Butanoate metabolism | 4 | 0.085 | 0.005 |
| ko00430 | Metabolism | Metabolism of other amino acids | Taurine and hypotaurine metabolism | 3 | 0.125 | 0.005 |
| ko04713 | Organismal Systems | Environmental adaptation | Circadian entrainment | 2 | 0.222 | 0.007 |
| ko04080 | Environmental Information Processing | Signaling molecules and interaction | Neuroactive ligand-receptor interaction | 4 | 0.076 | 0.008 |
| ko04216 | Cellular Processes | Cell growth and death | Ferroptosis | 3 | 0.103 | 0.009 |
| ko04978 | Organismal Systems | Digestive system | Mineral absorption | 3 | 0.103 | 0.009 |
| ko04070 | Environmental Information Processing | Signal transduction | Phosphatidylinositol signaling system | 3 | 0.103 | 0.009 |
| ko01232 | Metabolism | Global and overview maps | Nucleotide metabolism | 4 | 0.069 | 0.011 |
| ko04072 | Environmental Information Processing | Signal transduction | Phospholipase D signaling pathway | 2 | 0.182 | 0.011 |
| ko04917 | Organismal Systems | Endocrine system | Prolactin signaling pathway | 2 | 0.182 | 0.011 |
| ko04742 | Organismal Systems | Sensory system | Taste transduction | 3 | 0.094 | 0.012 |
| ko04728 | Organismal Systems | Nervous system | Dopaminergic synapse | 2 | 0.167 | 0.013 |
| ko07211 | Drug Development | Target-based classification: G protein-coupled receptors | Serotonin receptor agonists/antagonists | 1 | 1.000 | 0.015 |
| ko04750 | Organismal Systems | Sensory system | Inflammatory mediator regulation of TRP channels | 3 | 0.086 | 0.015 |
| ko05014 | Human Diseases | Neurodegenerative disease | Amyotrophic lateral sclerosis | 2 | 0.143 | 0.018 |
| ko01063 | Metabolism | Chemical structure transformation maps | Biosynthesis of alkaloids derived from shikimate pathway | 6 | 0.042 | 0.020 |
| ko00460 | Metabolism | Metabolism of other amino acids | Cyanoamino acid metabolism | 3 | 0.067 | 0.029 |
| ko01055 | Metabolism | Metabolism of terpenoids and polyketides | Biosynthesis of vancomycin group antibiotics | 2 | 0.105 | 0.032 |
| ko00120 | Metabolism | Lipid metabolism | Primary bile acid biosynthesis | 3 | 0.064 | 0.033 |
| ko00562 | Metabolism | Carbohydrate metabolism | Inositol phosphate metabolism | 3 | 0.064 | 0.033 |
| ko00260 | Metabolism | Amino acid metabolism | Glycine, serine and threonine metabolism | 3 | 0.063 | 0.034 |
| ko00380 | Metabolism | Amino acid metabolism | Tryptophan metabolism | 4 | 0.048 | 0.035 |
| ko00360 | Metabolism | Amino acid metabolism | Phenylalanine metabolism | 3 | 0.061 | 0.036 |
| ko02020 | Environmental Information Processing | Signal transduction | Two-component system | 3 | 0.057 | 0.044 |
| ko00965 | Metabolism | Biosynthesis of other secondary metabolites | Betalain biosynthesis | 2 | 0.087 | 0.046 |
